# Advances in Understanding Mating Type Gene Organization in the Mushroom-Forming Fungus *Flammulina velutipes*

**DOI:** 10.1534/g3.116.034637

**Published:** 2016-09-09

**Authors:** Wei Wang, Lingdan Lian, Ping Xu, Tiansheng Chou, Irum Mukhtar, Aron Osakina, Muhammad Waqas, Bingzhi Chen, Xinrui Liu, Fang Liu, Baogui Xie, Arend F. van Peer

**Affiliations:** * Mycological Research Center, College of Life Sciences, Fujian Agriculture and Forestry University, Fuzhou 350002, China; † Department of Fungal Resource, College of Plant Protection, Shandong Agricultural University, Tai’an 271018, China; ‡ College of Crop Sciences, Fujian Agriculture and Forestry University, Fuzhou 350002, China; § College of Food Sciences, Fujian Agriculture and Forestry University, Fuzhou 350002, China

**Keywords:** mating type, homeodomain, pheromone receptor, *Flammulina velutipes*, Genetics of Sex

## Abstract

The initiation of sexual development in the important edible and medicinal mushroom *Flammulina velutipes* is controlled by special genes at two different, independent, mating type (MAT) loci: HD and PR. We expanded our understanding of the *F. velutipes* mating type system by analyzing the MAT loci from a series of strains. The HD locus of *F. velutipes* houses *homeodomain* genes (*Hd* genes) on two separated locations: sublocus HD-a and HD-b. The HD-b subloci contained strain-specific *Hd1*/*Hd2* gene pairs, and crosses between strains with different HD-b subloci indicated a role in mating. The function of the HD-a sublocus remained undecided. Many, but not all strains contained the same conserved *Hd2* gene at the HD-a sublocus. The HD locus usually segregated as a whole, though we did detect one new HD locus with a HD-a sublocus from one parental strain, and a HD-b sublocus from the other. The PR locus of *F. velutipes* contained *pheromone receptor* (*STE3*) and *pheromone precursor* (*Pp*) genes at two locations, sublocus PR-a and PR-b. PR-a and PR-b both contained sets of strain-specific *STE3* and *Pp* genes, indicating a role in mating. PR-a and PR-b cosegregated in our experiments. However, the identification of additional strains with identical PR-a, yet different PR-b subloci, demonstrated that PR subloci can recombine within the PR locus. In conclusion, at least three of the four MAT subloci seem to participate in mating, and new HD and PR loci can be generated through intralocus recombination in *F. velutipes*.

Sexual development in basidiomycetous fungi is controlled by specialized genes that are located at distinct loci called mating type (MAT) loci (Raper 1966; Brown and Casselton 2001; Raudaskoski and Kothe 2010; Raudaskoski 2015). In the majority of mushroom-forming fungi (homobasidiomycetes), two different and unlinked MAT loci can be found: MAT-A and MAT-B. Recently, MAT-A and MAT-B loci were named HD and PR loci, respectively (Kües 2015; Maia *et al.* 2015). Upon mating, the two parental sets of HD and PR loci can recombine into four sexually distinct mating types—a system referred to as the tetrapolar mating type system. The origin of the tetrapolar mating type system has been argued to be ancient for basidiomycetes (Burnett 1975; Fraser *et al.* 2007; Hsueh and Heitman 2008; Maia *et al.* 2015), and the genomes of tetrapolar, as well as bipolar and homothallic mushroom-forming species are known to contain both the HD and the PR locus (James *et al.* 2006; Aimi *et al.* 2005; Yi *et al.* 2009; Morin *et al.* 2012; Bao *et al.* 2013; Chen *et al.* 2016). Further developments in mushroom-forming fungi have resulted in often elaborate HD and PR systems that consist of multiple HD and PR subloci, each containing a series of possible alleles (Casselton and Olesnicky 1998; James *et al.* 2004b; Whitehouse 1949; Raper 1966; Casselton 1978; Badrane and May 1999).

The basic model of the HD locus encompasses a divergently transcribed pair of *homeodomain1* (*Hd1*) and *homeodomain2* (*Hd2*) genes that form a functional group, flanked by the *mitochondrial intermediate peptidase* (*MIP*) gene on one side, and the *β-flanking* gene on the other (James *et al.* 2004a; Kües *et al.* 2011). In the case of multiple HD subloci, several of these functional groups will be positioned between the *MIP* gene and the *β-flanking* gene (Brown and Casselton 2001; James 2007; Kües *et al.* 2011). The products encoded by the *Hd1* and *Hd2* gene pairs interact exclusively with HD proteins encoded by different alleles (nonself) of the same sublocus (Kües and Casselton 1992; Kües *et al.* 1994; Banham *et al.* 1995). HD subloci can be functionally redundant, and one compatible *Hd1* and *Hd2* gene are sufficient to mediate activation of the HD pathway. Upon interaction, an HD1 and HD2 protein will form a heterodimeric transcription factor that activates the sexual developmental pathway under the control of the HD locus (Brown and Casselton 2001). While the characteristic DNA binding motifs (the HD1 or HD2 domain) of the HD proteins are mostly conserved, allelic variation and mating compatibility is determined by the highly variable N-terminal regions (Kronstad and Leong 1990; Yee and Kronstad 1993; Banham *et al.* 1995; Wu *et al.* 1996; Badrane and May 1999; Coelho *et al.* 2010). In addition, divergence of the C-terminal parts of the HD-encoding genes has been argued to be useful in preventing the recombination of alleles on the same sublocus, which could otherwise result in self-activation (for a discussion see Kües 2015, and references herein). The HD locus and the surrounding genomic region are highly syntenic in most mushroom-forming fungi, and synteny is often maintained when gene clusters or HD subloci are transferred to other positions in the genome (James *et al.* 2006; Niculita-Hirzel *et al.* 2008; Bao *et al.* 2013; van Peer *et al.* 2011).

The basic PR locus consists of an archetypal subunit that contains a single *pheromone receptor* gene (*STE3*) in association with one or several *pheromone precursor* (*Pp*) genes (Vaillancourt *et al.* 1997; Casselton and Olesnicky 1998; O’Shea *et al.* 1998; Halsall *et al.* 2000; Casselton and Challen 2006; James 2007). Multiple of these subunits can form PR subloci. Pheromone receptors and pheromones within a subunit are incompatible, and only interact with products of different allelic subunits from corresponding subloci. PR subloci can be functionally redundant, yet not all nonself combinations are always active. In case of a compatible interaction, a pheromone peptide will bind the extracellular domain of a pheromone receptor, triggering activation of the PR pathway (Olesnicky *et al.* 2000; Brown and Casselton 2001). The fungal pheromone receptors are class D, G-protein coupled receptors that belong to the Ste3 subfamily (GPCRs, http://pfam.xfam.org/family/PF02076; Finn *et al.* 2016). They contain seven transmembrane (7-TM) domains, extracellular N-termini, and intracellular C-terminal regions (Dohlman *et al.* 1991; Caldwell *et al.* 1995). Pheromone precursors encode 40–100 amino acid long prepeptides. They mature through farnesylation of the C-terminal CAAX motif, and presumed splicing at a conserved position with two charged amino acids (*e.g.*, “ER,” “ED,” or “EH”) located 10–15 amino acids upstream of the C-terminus (Bölker and Kahmann 1993; Caldwell *et al.* 1995; O’Shea *et al.* 1998; Fowler *et al.* 2001; Kües 2015). The PR loci and the surrounding genomic region are poorly conserved (James 2007), and PR subloci can be separated by distances of up to 170 kb (van Peer *et al.* 2011).

In addition to *Hd*, *STE3*, and *Pp* genes, many mushroom-forming fungi contain *pheromone receptor like* genes, which share considerable sequence similarity with the *pheromone receptor* genes on the PR loci (Niculita-Hirzel *et al.* 2008; van Peer *et al.* 2011, Kües 2015). Yet, *pheromone receptor like* genes are conserved (there are no different alleles in different mating types), and are not associated with *pheromone precursor* genes (Niculita-Hirzel *et al.* 2008; van Peer *et al.* 2011). To determine which genes belong to PR (sub)loci, and which PR and HD subloci partake in mating, it is necessary to identify and compare the PR and HD loci of different mating types. *Flammulina velutipes* (Curt. ex Fr.) Sing, also known as Winter Mushroom or Enokitake, is an important edible and medicinal mushroom that is cultivated on a large scale (Tonomura 1978; Leifa *et al.* 2001; Wang *et al.* 2012; Zhang *et al.* 2012; Sekiya *et al.* 2013). *F. velutipes* has a tetrapolar mating type system that generates basidiospores of four possible mating types. Although single mating types have been known to produce fruiting bodies under severe stress, they are typically self-infertile and controlled by the HD and PR pathways (Uno and Ishikawa 1971; Esser *et al.* 1979; Wessels 1993; Yi *et al.* 2007). The first HD and PR loci of *F. velutipes* were identified recently, indicating that the HD locus consisted of two subloci, HD-a and HD-b, and that the PR locus also consisted of two subloci, PR-a and PR-b (van Peer *et al.* 2011). As few mating compatible HD or PR loci have been identified, it remained unclear if (a) the reported organization of the HD and PR subloci was representative for *F. velutipes*, and (b) which of these subloci participated in mating. Only PR-a had been deduced to be involved in mating. In this study, we identified and compared the structure and content of a series of HD and PR subloci, using whole genome sequences as well as subcloned regions from a variety of *F. velutipes* strains.

## Materials and Methods

### Strains and media

Monokaryotic *F. velutipes* strains W23 (A2B5) and L11 (A1B1), dikaryotic strain 1123 (a cross of strain L11 and strain W23), and *F. velutipes* strains F0010, F0012, F0020, F0027, and F0025 (Jiang *et al.* 2009), were provided by the Fujian Edible Fungi Germplasm Resource Collection Center of China (Supplemental Material, Table S1). All strains were maintained on Potato Dextrose Agar medium (PDA; 200 g/l potato; 20 g/l glucose; 20 g/l agar) at 25°.

To produce fruiting bodies for spore isolation, strains were cultivated in sterile plastic bags containing growth substrate (cottonseed hulls 52.5%, wheat bran 25%, sawdust 15%, corn flour 5%, gypsum 2%, and ground limestone 0.5%, with a moisture content of 61%). Strains were grown at 23° for 30 d, followed by cold-stimulation at 16° and 90% humidity until primordia occurred (1 wk). Cultures were maintained at low temperature (16° and 75% humidity) to allow full fruiting body development.

For single-spore cultures, spores were collected by placing a paper under the pilei of harvested, mature fruiting bodies. Spores were washed with sterile ddH_2_O, inoculated on PDA, and incubated at 25° for 24–48 hr until spore germination occurred. Single spore isolates (SSIs) were selected under an optical microscope (Motic) and subcultured individually on PDA at 25°.

For DNA extraction, mycelium of *F. velutipes* strains was grown for 2 wk at 25° on PDA overlaid with a cellophane membrane. Harvested mycelium was stored at −80° until use.

### Genome and transcriptome sequences of F. velutipes strains

The genome sequence of strain KACC42780 (Park *et al.* 2014) was provided by W.S. Kong of the Rural Development Administration (RDA) in Korea. Genome sequences of *F. velutipes* strain L11 and strain W23 were obtained from our lab (Zeng *et al.* 2015; Liu *et al.* 2015; Wang *et al.* 2015b). Genomic DNA was extracted from mycelium using a CTAB method (Stajich *et al.* 2010). A paired-end (2 × 90 bp) DNA library with 500 bp inserts was generated and sequenced (*de novo* sequencing) on a Illumina Cluster Station and Illumina GAII platform by Zhejiang California International NanoSystems Institute (Hangzhou, China). Over 6 GB clean reads were obtained for the genomes of strain L11 and W23, amounting to ∼170× coverage.

For transcriptomics, mycelium of monokaryotic strains L11 and W23, and dikaryotic strain 1123, was collected and flash frozen in liquid nitrogen. RNA was extracted and used for library construction (length of paired-end reads, 2 × 90 bp; insert size, 500 bp), and sequencing (Illumina Cluster Station, Illumina GAII platform, http://www.genomics.cn) at BGI (Shenzhen, China) (Liu *et al.* 2014; Wang *et al.* 2015a). Transcriptomics data were used to correct intron-exon boundaries of predicted gene models by using ZOOMlite software (Lin *et al.* 2008; Zhang *et al.* 2010).

### Identification of mating type genes and analysis of predicted proteins

The genomes of *F. velutipes* strain L11 and W23 were screened for mating type genes using BLASTP 2.2.26 homology searches (Altschul *et al.* 1997) with known *F. velutipes* mating type proteins (van Peer *et al.* 2011). Gaps in the sequences of newly found mating type genes were filled by sequencing of PCR segments (Sangon, Shanghai, China). *Pheromone precursor* genes were identified as described before (van Peer *et al.* 2011).

Nuclear localization signals (NLS), dimerization motifs (Di), and homeodomains (HD) of homeodomain proteins were predicted by PSORT II Prediction (http://psort.hgc.jp/form2.html; Horton *et al.* 2007), COILS Server (http://embnet.vital-it.ch/software/COILS_form.html; Windows width = 14; Lupas *et al.* 1991), and MOTIF Search (http://www.genome.jp/tools/motif/). Transmembrane helices of pheromone receptors were predicted with the TMHMM Server (http://www.cbs.dtu.dk/services/TMHMM-2.0/) and the TMpred Server (http://www.ch.embnet.org/software/TMPRED_form.html). Multiple sequence alignments of the HD and PR mating type genes and subloci were performed with Clustal Omega (http://www.ebi.ac.uk/Tools/msa/clustalo/). Synteny maps were drawn using Chromomapper 1.0.10.26 (Niculita-Hirzel and Hirzel 2008).

### Phylogenetic analysis of pheromone receptors and pheromone-receptor-like proteins

Pheromone receptors and pheromone-receptor-like proteins of *Laccaria bicolor* (Niculita-Hirzel *et al.* 2008), *Schizophyllum commune*, *Pleurotus djamor*, *Coprinopsis cinerea*, *Cryptococcus neoformans* (James *et al.* 2004b), together with pheromone receptors CcRcb2.43 (GenBank accession number: AAQ96345), CcRcb2.44 (GenBank accession number: AAQ96344), and ScBar8 (GenBank accession number: AAR99618) were selected for multiple alignment and tree building using the online tool “Phylogeny.fr” with “One click” stream (http://phylogeny.lirmm.fr/phylo_cgi/index.cgi, Dereeper *et al.* 2008). In the first step, protein sequences were aligned using MUSCLE 3.7 (“Find diagonals” option disabled; Maximum number of iterations: 16, Edgar 2004). Next, ambiguous regions (*i.e.*, gapped and/or poorly aligned regions) were removed using Gblocks (v0.91b/minimum length of a block after gap cleaning was 10/no gap positions were allowed in the final alignment/all segments with contiguous nonconserved positions > 8 were rejected/minimum number of sequences for a flank position was 85%, Castresana 2000). Hereafter, the resulting 230 amino acids sequences (alignment of pheromone receptors and pheromone-receptor-like proteins of *F. velutipes* only), and 197 amino acids (alignment of pheromone receptors and pheromone-receptor-like proteins of multiple mushroom-forming fungi), were used to assemble phylogenetic trees. The supplemental materials file “Fv_STE3-Ori.txt” contains the original protein sequences, file “Fv_STE3-align_cured.txt” contains the processed sequences. Trees were reconstructed using maximum likelihood, as implemented in the PhyML program v3.0 (Guindon *et al.* 2010). The WAG substitution model was selected, assuming an estimated proportion of invariant sites and four gamma-distributed rate categories to account for rate heterogeneity across sites. The gamma shape parameter was estimated directly from the data. Reliability of internal branches was assessed using LRT testing (SH-Like) (Anisimova and Gascuel 2006). Graphical representation and editing of the phylogenetic tree were performed with TreeDyn v198.3 (Chevenet *et al.* 2006).

### Crossing of strains and segregation analysis of mating-type genes

Genomic DNA from *F. velutipes* SSIs was extracted as described (van Peer *et al.* 2011). Mating type gene-specific primers (Table S2A) were used to determine the presence or absence of these genes. PCR was performed in volumes of 30 µl premix *Taq*kit (Takara, China), using 35 cycles and an initial melting step of the genomic DNA at 94° for 5 min. To establish the site of recombination between the HD-a and HD-b subloci, specific primers were designed that flanked single nucleotide polymorphisms (SNPs) located between the HD-a and HD-b subloci in strain L11 and W23 (Table S2B).

SSIs of *F. velutipes* strains F0010, F0012, F0020, F0025, and F0027 (Table S1) were crossed to distinguish the four different mating types of each strain, followed by PCR amplification (primers: Table S2C), and sequencing of the genes at the respective HD-a and HD-b subloci (Table S3). Compatibility of HD-b subloci was determined by crossing *F. velutipes* strain L11 with other strains containing different HD-b and similar HD-a subloci.

### Data availability

The data from this study were deposited in NCBI’s GenBank under the accession numbers: HQ630588.1, HQ630589.1, HQ630597.1, HQ630590.1, HQ630591.1, HQ630592.1, HQ630593.1, HQ630594.1, HQ630595.1, HQ630596.1, BK009409.1, KC208604.1, KC208603.2, KC208605.1, KC208611.1, KC208606.2, KC208607.1, KC208608.2, KC208609.2, KC208610.1, KT808674.1, KC208594.1, KC208595.2, KC208612.1, KC208596.1, KC208597.1, KC208598.2, KC208599.1, KC208600.2, KC208601.2, KC208602.1, KT808675.1, KT808673.1, KT808672.1, KT808676.1, KT808677.1, KT808678.1 and KT808679.1. Genome sequences have been deposited at DDBJ/EMBL/GenBank (http://www.ncbi.nlm.nih.gov/) under accession nos. APHZ00000000 (W23; BioProject: 191864) and APIA00000000 (L11; BioProject: 191865). Transcriptomics data has been uploaded to the NCBI Sequence Read Archive (SRA), and is accessible through accession numbers: SRP072148–SRP072150 (http://www.ncbi.nlm.nih.gov/sra/). 

## Results

### Identification of mating type genes in F. velutipes strain L11, W23, and KACC42780

New HD and PR loci of *F. velutipes* were identified using the draft genomes of two mating-compatible strains; *F. velutipes* strain L11 and *F. velutipes* strain W23 (Liu *et al.* 2015; Wang *et al.* 2015b; Zeng *et al.* 2015). The draft genome of strain L11 consisted of 1858 scaffolds covering 34.75 Mb and 11,526 predicted genes. The draft genome of strain W23 consisted of 1866 scaffolds, covering 35.13 Mb and 11,071 predicted genes (Table S4 and Table S5). A complete list of the identified mating type genes and their gene accession numbers is given (Table S3).

**Figure 1:**
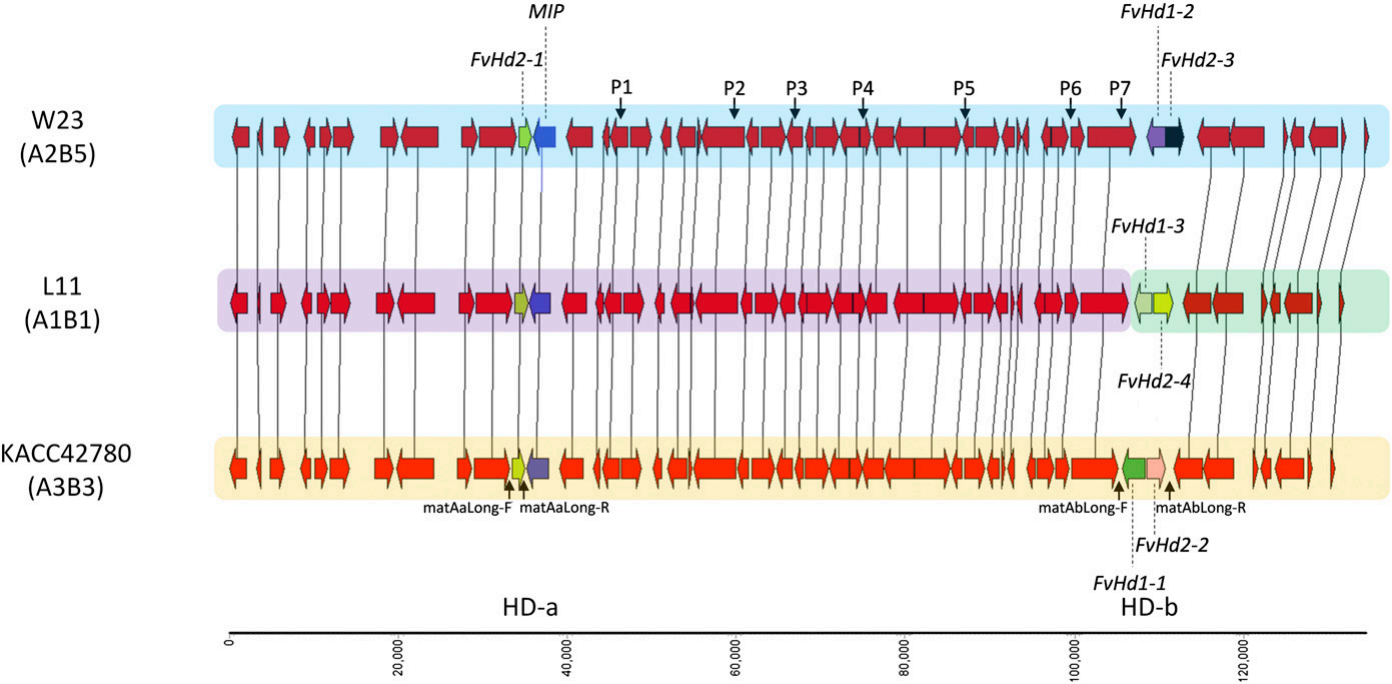
Synteny map of the HD loci of *F. velutipes* strains W23, L11, and KACC42780. Names and mating types of strains are indicated at the left, the relative size of the regions is indicated in nucleotides at the bar at the bottom. The *Mitochondrial Intermediate Peptidase* (*MIP*) gene (blue arrow), and the different *Hd* genes (green, yellow, purple, and black arrows) are indicated separately. Locations of the primer sets that were used to determine the site of recombination between the HD-a and the HD-b sublocus in SSI No.24 are indicated with P1–P7. The binding sites for the conserved primers that were used to amplify the HD-a and HD-b subloci of *F. velutipes* strains are indicated under the HD region of strain KACC42780.

To identify HD loci, the genomes of strain L11 and strain W23 were screened for *homeodomain* genes based on homology searches with *F. velutipes* homeodomain proteins FvHD1-1, FvHD2-1, and FvHD2-2 (van Peer *et al.* 2011). Strain L11 contained three *homeodomain* genes, one *FvHd2* gene on scaffold 1208, and an *FvHd2*/*FvHd1* gene pair on scaffold 336 (Figure 1). The genome of strain W23 contained three *homeodomain* genes on scaffold 63, one single *FvHd2* gene, and an *FvHd2*/*FvHd1* gene pair (Figure 1). Reciprocal BLAST searches with the newly identified *FvHd* genes did not reveal any additional *homeodomain* genes in the genomes of strain L11, W23, or in the genome of strain KACC42780.

**Figure 2:**
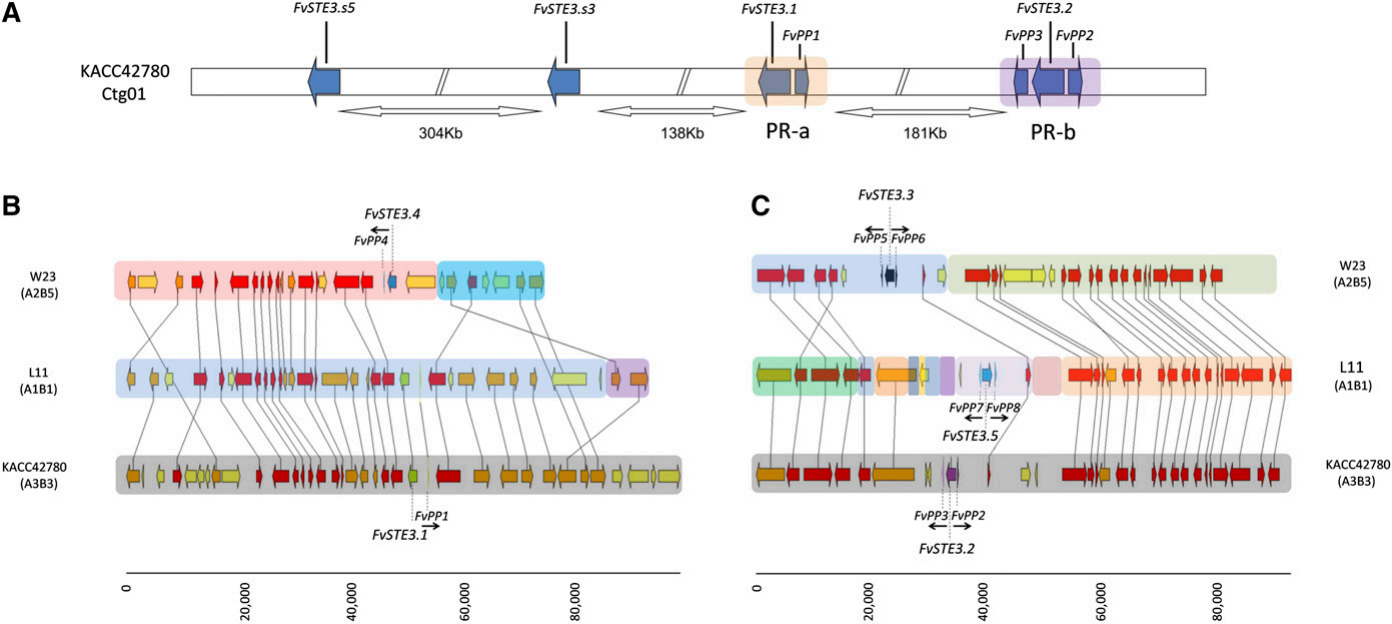
(A) General organization of the PR-a and PR-b subloci of *F. velutipes* according to the updated genome sequence (Park *et al.* 2014). *Pheromone receptor* genes (*FvSTE3.1* and *FvSTE3.2*), *pheromone precursor* genes (*FvPp1*, *FvPp2*, and *FvPp3*), and *pheromone receptor like* genes (*FvSTE3.s3* and *FvSTE3.s5*) are indicated. (B, C) Synteny map of respectively the PR-a (B) and PR-b (C) sublocus regions of *F. velutipes* strains W23, L11, and KACC42780. Strain names and their mating types are indicated at the left (B) or right (C) of the depicted regions. The positions and directions of *pheromone receptor* genes (*FvSTE3*, blue, green, purple, and black arrows), and *pheromone precursor* genes (*FvPP*, black arrows below or above the gene name) are depicted. The lower bar indicates the relative distance between genes in nucleotides.

For identification of PR loci, *pheromone receptor* genes were identified in the genomes of strain L11 and strain W23 based on homology searches with mating type specific pheromone receptor proteins FvSTE3.1 and FvSTE3.2 of *F. velutipes* strain KACC42780 (van Peer *et al.* 2011). *F. velutipes* strain L11 contained two *pheromone receptor* genes located on scaffolds 263 and 789 (Figure 2, B and C). Two *pheromone receptor* genes were also identified in the genome of strain W23, on scaffolds 1147 and 1065 (Figure 2, B and C). *Pheromone precursor* genes were identified through analysis of the flanking regions of the new *pheromone receptor* genes (van Peer *et al.* 2011). The *pheromone receptor* gene on the PR-a sublocus of strain L11 was accompanied by a single *pheromone precursor* gene (Figure 2B). The *pheromone receptor* gene on the PR-b sublocus of strain L11 was associated with two *pheromone precursor* genes (Figure 2C). Similarly, the *pheromone receptor* gene at the PR-a sublocus of strain W23 was associated with one *pheromone precursor* gene (Figure 2B), while the second *pheromone receptor* gene at the PR-b sublocus was associated with two *pheromone precursor* genes (Figure 2C).

Next, the genomes of strain L11 and W23 were screened for *nonmating type specific pheromone receptor* genes (*STE3.s*), hereafter called “*pheromone receptor like* genes” based on homology searches with five STE3.s proteins from *F. velutipes* strain KACC42780 (van Peer *et al.* 2011). All five *pheromone receptor like* genes (Table S3) were represented in the genomes of strain L11 (scaffolds 13, 188, 383, 409, and 1473), and strain W23 (scaffolds 247, 275, 353, 576, and 1302). In addition, a new and sixth *pheromone receptor like* gene was detected in the genomes of strain L11 and W23 (Table S3). This sixth *pheromone receptor like* gene was subsequently identified in the genome of strain KACC42780 (ctg11_2-8_draft).

### Analysis of homeodomain genes and proteins

**Figure 3:**
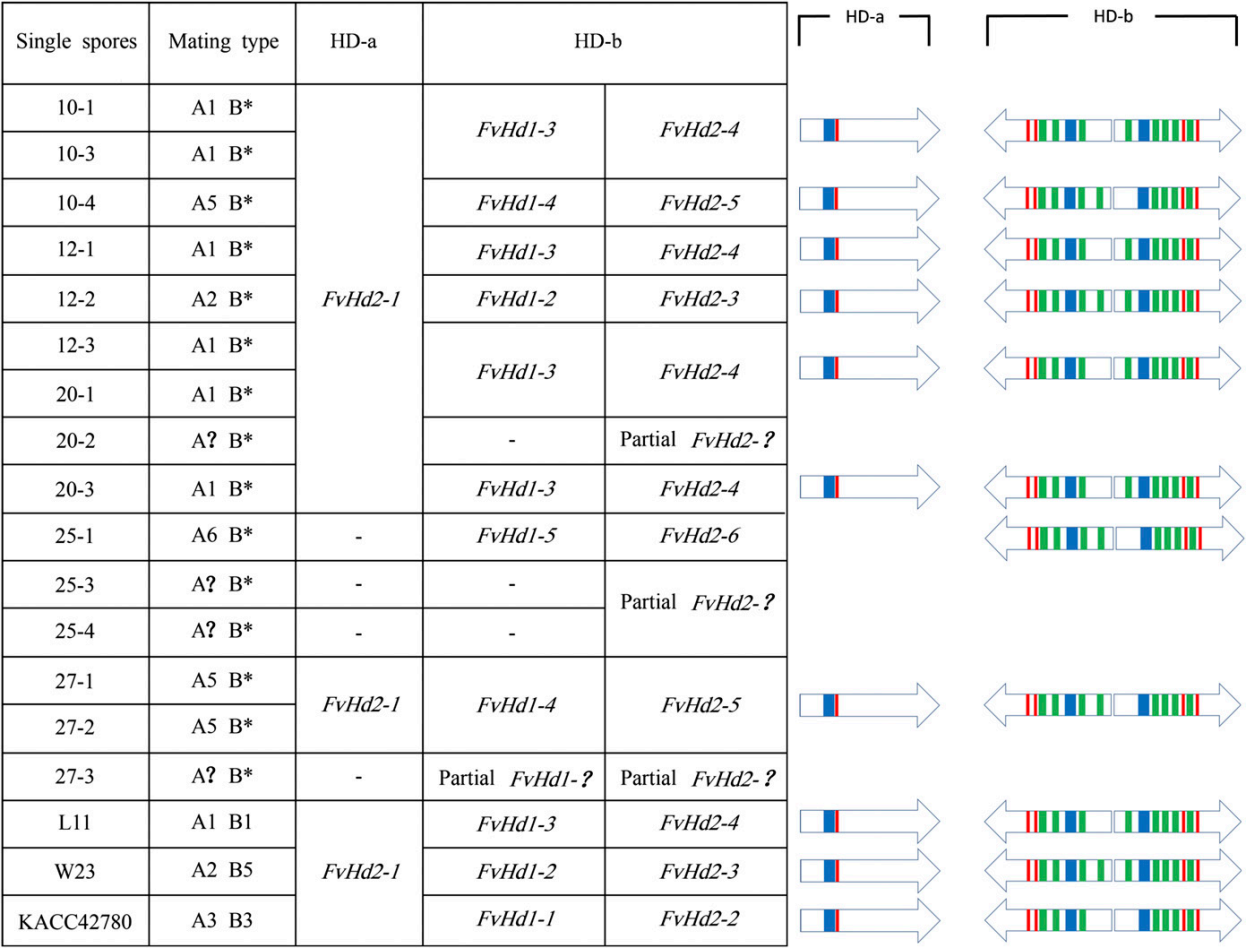
Mating types of strains L11, W23, KACC42780, and the SSIs of which HD subloci were determined by PCR. PR mating types are named in the second column when known, or indicated with (*) when unknown. Schematic representations of the corresponding HD proteins (arrows) indicated the orientation of the gene on each sublocus, the position of the homeodomains (blue), NLSs (red), and the Di motifs (green). HD-a subloci for which no PCR products could be obtained, and HD-b subloci for which the obtained PCR products could not be fully sequenced, are indicated with “−” and are not represented by a schematic drawing. HD mating types were assigned to the *F. velutipes* strains in agreement with van Peer *et al.* (2011), applying HD3 (previously A3) for strain KACC42780, and preserving “HD4” (previously A4) for strain KACC43777.

The predicted gene models (intron and exon boundaries) of the *homeodomain* genes of strain L11 and W23 were verified using transcriptomics data of strain L11, W23, and dikaryotic strain 1123. Alignment of the *homeodomain* genes showed that strain L11, W23, and KACC42780 contained the same *FvHd2-1* gene on the HD-a sublocus, with DNA sequence similarities of 99.77% over 1580 bp (Figure S1). The corresponding FvHD2-1 proteins showed 100% amino acid (AA) sequence identity. The FvHD2-1 proteins contained one predicted NLS. No Di motifs were detected in FvHD2-1 (Figure 3 and Figure S2). The *FvHd2-1* genes and proteins on the HD-a subloci were clearly dissimilar from the *FvHd2* genes and proteins on the HD-b subloci (Figure S2). DNA sequence alignment of the *homeodomain* genes on the HD-b subloci of strain L11, W23, and KACC42780 indicated that each strain contained a pair of unique *FvHd1*/*FvHd2* genes (Figure S3, Figure S4, and Figure S5). DNA sequence polymorphism was restricted to the N-terminal portions of the *FvHd1* and *FvHd2* genes, while the remaining parts were highly conserved. The short intergenic region between the *FvHd1* and *FvHd2* genes on the HD-b subloci also revealed high DNA sequence polymorphism. In accordance with the DNA sequences, the N-termini of the FvHD1 and FvHD2 proteins on the HD-b sublocus were strongly dissimilar (Figure S6 and Figure S7). The remaining four-fifths of the total protein lengths of the HD proteins were conserved. These included most of the Di and NLS motifs (Figure 3). The FvHD1 and FvHD2 proteins on the HD-b sublocus of strain KACC42780 did not contain predicted Di motifs in the variable N-termini, in contrast to the FvHD1 and FvHD2 proteins on the HD-b subloci of strain L11 and strain W23. The FvHD2 proteins of the HD-b subloci of strain KACC42780 (688 AA), L11 (688 AA), and W23 (693 AA) were longer than the FvHD2-1 proteins (475 AA) of the HD-a sublocus. Conservation between the HD proteins from strain L11, W23, KACC42780, and the HD proteins of additionally identified HD-b subloci (see below) was high (Figure S8 and Figure S9).

The strong conservation of FvHD1 and FvHD2 proteins at the HD-b subloci in *F. velutipes* (86.06–89.81%) was unexpected. Interestingly, comparison of HD1 and HD2 sequences from other mushrooms, of which multiple alleles of HD subloci had been reported, revealed large variations in the conservation levels of these proteins. HD1 and HD2 proteins of *Lentinula edodes* (Au *et al.* 2014) showed high similarity (76.46–77.9%, Figure S10 and Figure S11). In contrast, HD1 and HD2 proteins in *S. commune* showed considerably lower similarity (38.85–58.89%, Figure S12, Figure S13, and Figure S14), while HD1 and HD2 proteins of different subloci in *C. cinerea* (Badrane and May 1999) showed strongly varying conservation levels, depending on the sublocus (39.26–70.3%, Figure S15 and Figure S16).

### Comparison of the HD subloci and genomic regions

The genomic region containing the HD subloci is generally conserved in mushroom-forming fungi. In *F. velutipes*, this region had been indicated to contain structural deviations from the conserved gene arrangement (van Peer *et al.* 2011). To verify these differences, we compared the HD-a and HD-b sublocus-containing regions of strain L11 and W23, with that of the latest version of the *F. velutipes* KACC42780 genome that consisted of 11 chromosomes (35.6 Mb, 12,218 predicted genes; Park *et al.* 2014). The *homeodomain* genes of strain KACC42780 were located on chromosome 8 (2011_ctg03_draft). The HD-a and HD-b subloci on chromosome 8 were confirmed to be separated by ∼70 kb as previously reported, and no *β-flanking* gene was present near the HD-a or HD-b subloci. Alignment of a 134 kb section of chromosome 8 of strain KACC42780, with scaffold 63 of strain W23, and scaffolds 1208 and 336 of strain L11, revealed absolute synteny (Figure 1). The three HD-a subloci of strain L11, W23 and KACC42780, each contained the same single *FvHd2-1* gene, without an associated *FvHd1* gene. The HD-a sublocus was flanked by the *MIP* gene, which was located between the HD-a and the HD-b sublocus (Figure 1). All three HD-b subloci contained a pair of similarly arranged *FvHd1* and *FvHd2* genes.

### Identification of additional HD subloci

Previously, the HD-a sublocus of strain KACC43777 had been reported not to contain the conserved *FvHd2-1* gene. To further explore allelic variation of the HD subloci in *F. velutipes*, SSIs of five dikaryotic strains were selected for cloning and analysis of their respective HD-a and HD-b subloci (Figure 3 and Table S1). To ensure that all possible HD loci of the five strains were included, three SSIs with different mating types were analyzed from each dikaryon (15 in total). Specific primers were designed for the HD-a and the HD-b subloci, using flanking regions of the HD subloci that were conserved between the genomes of strain L11, W23, and KACC42780 (Figure 1 and Table S2C). Of the 15 SSIs that were analyzed, 11 contained a copy of the conserved *FvHd2-1* gene found at the HD-a sublocus in strain L11, W23, and KACC42780 (Figure 3 and Figure S1). Complete sequences of HD-b subloci were obtained for 11 SSIs as well. Six SSIs contained the same HD-b sublocus as strain L11, and one contained the same HD-b sublocus as strain W23. Three SSIs contained a new HD-b sublocus with an *FvHd1-4*/*FvHd2-5* gene pair, and one SSI contained a new HD-b sublocus with an *FvHd1-5*/*FvHd2-6* gene pair (Figure 3, Figure S4, and Figure S5). The HD-b subloci of the four remaining SSIs were subcloned successfully, yet their sequences could be only partially determined. It remained unknown if the *FvHd* genes at these HD-b subloci were different from already identified genes, due to the high conservation of the parts of the *FvHd* genes that were represented by the partial sequences. Overall, allelic variation was strongest at the HD-b sublocus. However, the absence of *FvHd2-1* in four SSIs might indicate variation (*i.e.*, no gene or a different gene instead of the *FvHd2-1* gene) at the HD-a sublocus.

### Analysis of pheromone receptor, pheromone receptor like, and pheromone precursor genes and their proteins

The predicted gene models of the *pheromone receptor* genes and *pheromone receptor like* genes of strain L11 and W23 were verified based on transcriptomics data of strain L11, W23, and dikaryotic strain 1123. Comparisons with the genes of KACC42780 revealed a few differences in the intron and exon boundaries of gene *FvSTE3.s2* of KACC42780 (van Peer *et al.* 2011). The gene model of *FvSTE3.s2* from strain KACC42780 was updated following the transcriptomics data of strain L11, W23, and dikaryotic strain 1123. TMHMM and TMpred prediction software showed that proteins FvSTE3.1 to FvSTE3.5 contained 7-TM domains that are characteristic of pheromone receptors (Table S6). Alignment of the *pheromone receptor* genes and *pheromone precursor* genes revealed that strain L11 and strain KACC42780 contained the same genes on the PR-a sublocus. The pheromone that was encoded by the *FvPp1* gene at the PR-a sublocus of KACC42780 had previously been reported to contain an additional tryptophan (W) behind the C-terminal CAAX box (CAAXW*). The *FvPp1* gene of strain L11 also encoded this additional tryptophan (Figure S17). Subcloning and resequencing of the *FvPp1* gene (primers Table S2D) confirmed that the sequence indeed encoded the extra tryptophan. The additional tryptophan was further supported by the transcriptomics data of strain L11, W23, and 1123 (Figure S18). Strain W23 contained different *pheromone receptor* (*FvSTE3.4*) and *pheromone precursor* (*FvPp4*) genes on the PR-a sublocus (Figure 2). Interestingly, the predicted mature FvPP4 pheromone on the PR-a sublocus of W23 (ERHGQGMAYTC), was identical to predicted mature pheromone FvPP1 except for the additional tryptophan at the C-terminus of FvPP1 (Figure S17). The *pheromone receptor* genes and *pheromone precursor* genes on the PR-b subloci of strain L11, W23, and KACC42780 were all strain specific (Figure 2). However, the mature FvPP3 pheromone from the PR-b sublocus of strain KACC42780, and the mature FvPP6 pheromone from the PR-b sublocus of strain W23, contained identical protein sequences (ERAGDPTFRGGAC). This indicated that pheromones FvPP3 and FvPP6 would be incompatible with pheromone receptors FvSTE3.2 and FvSTE3.3, respectively (Figure S17). Pheromone receptor like proteins FvSTE3.s1–FvSTE3.s6, which had been identified in the genomes of strain L11, W23 and KACC42780, each contained 7-TM domains (Table S6). None of the corresponding *pheromone receptor like* genes was accompanied by a *pheromone precursor* gene.

### Phylogenetic analysis of pheromone receptors and pheromone receptor like proteins

**Figure 4:**
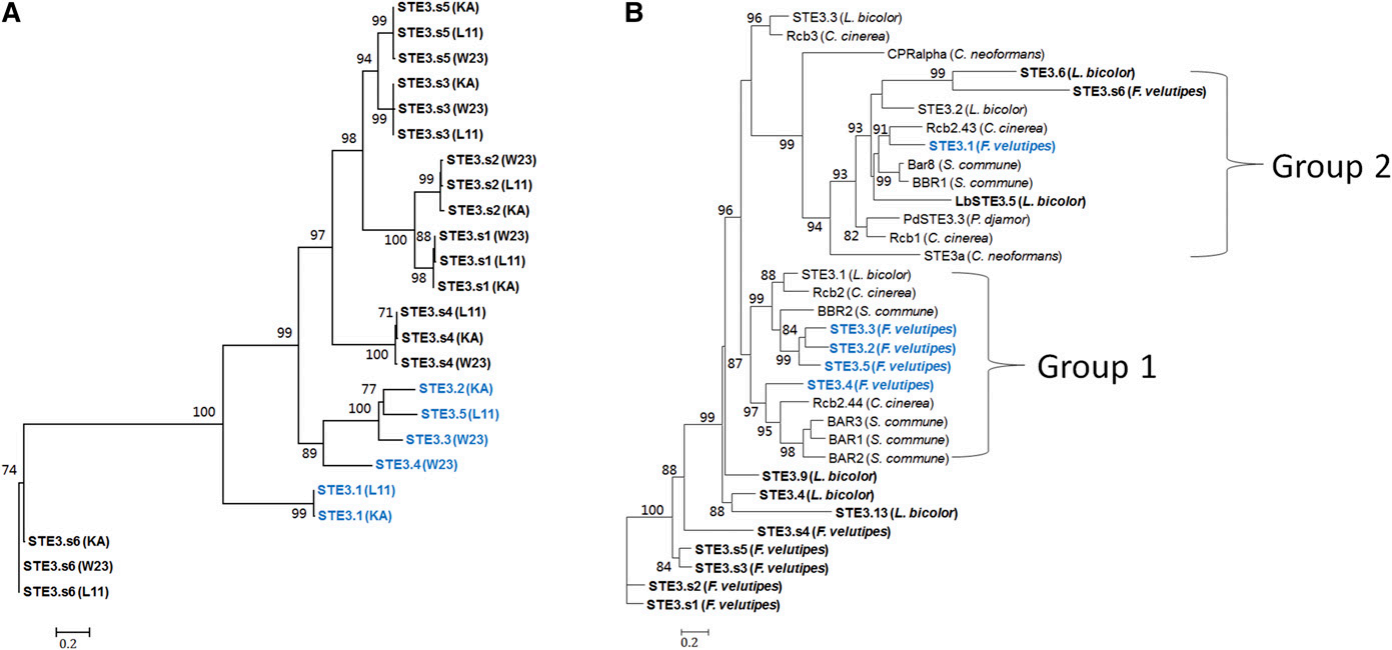
(A) Phylogenetic tree of the pheromone receptors (blue) and pheromone-receptor-like proteins (black) of *F. velutipes* strains L11, W23, and KACC42780. The corresponding strain is indicated behind each protein. (B) Phylogenetic tree of *F. velutipes* (blue) and *S. commune*, *C. cinerea*, *L. bicolor*, and *C. neoformans* (black) pheromone receptors. *F. velutipes* and *L. bicolor* pheromone-receptor-like proteins are given in black, bold font. Species names are indicated behind each protein. KA, strain KACC42780.

The phylogenetic origin of pheromone receptors and pheromone-receptor-like proteins has remained confused, and pheromone receptors from the same sublocus do not always cluster together (Riquelme *et al.* 2005; Kües 2015). To examine the relationship between the pheromone receptors and the pheromone-receptor-like proteins in *F. velutipes*, two analyses were performed. The first analysis comprised a comparison of all identified pheromone receptor and pheromone-receptor-like protein sequences from *F. velutipes* (Figure 4A). The second analysis compared the pheromone receptor and pheromone-receptor-like protein sequences from *F. velutipes* with that of other model mushroom-forming fungi (Figure 4B). Pheromone receptor and pheromone-receptor-like protein sequences from *F. velutipes* that were represented by multiple copies, like, for example, the FvSTE3.s proteins, were included only once in the second analysis. In brief, proteins were aligned using MUSCLE, and processed with Gblocks to remove poorly aligned regions. The resulting 230 amino acid sequences (first analysis), and 197 amino acid sequences (second analysis), were compiled into a maximum likelihood tree. For both analyses, the graphics of the resulting trees were adjusted with TreeDyn. The pheromone receptors of *F. velutipes* were divided into two main groups (Figure 4A). Comparison with other fungal pheromone receptors placed FvSTE3.1 in main group “Group2,” and FvSTE3.2 to FvSTE3.5 in main group “Group1” (Figure 4B). Pheromone receptors FvSTE3.2, FvSTE3.3, and FvSTE3.5 were closely related, in accordance with their localization on the same PR-b sublocus. FvSTE3.4 appeared less related to FvSTE3.2, FvSTE3.3, and FvSTE3.5 (Figure 4A). This separation was confirmed by comparison of the pheromone receptors from different fungi (Figure 4B). It is interesting to note that FvSTE3.4 is located on the PR-a sublocus, like FvSTE3.1, instead of on the PR-b sublocus with FvSTE3.2, FvSTE3.3, and FvSTE3.5. The presence of two pheromone receptors from different main phylogenetic groups (*i.e.*, FvSTE3.1 and FvSTE3.4) on the same PR-a sublocus indicated that recombination of pheromone receptors from different subloci must have occurred. The pheromone-receptor-like proteins clustered in two groups that were clearly separated from the pheromone receptors of *F. velutipes*. One small group contained the FvSTE3.s6 proteins, and a second, larger group contained the FvSTE3.s1 to FvSTE3.s5 proteins (Figure 4A). Within the larger group of pheromone-receptor-like proteins, FvSTE3.s1 and FvSTE3.s2 were indicated to be related more closely, as were FvSTE3.s3 and FvSTE3.s5 (Figure 4A). When compared to other fungal pheromone receptors and pheromone-receptor-like proteins, FvSTE3.s1 to FvSTE3.s5 remained distant from the main pheromone receptor groups “Group1” and “Group2” (Figure 4B). No pheromone-receptor-like proteins clustered in pheromone receptor main group “Group1.” However, FvSTE3.s6 was included in pheromone receptor main group “Group2,” together with pheromone-receptor-like proteins from *L. bicolor* (STE3.5 and STE3.6).

### Comparison and analysis of PR loci

The PR loci of strain L11 and W23 were compared with the latest version of the *F. velutipes* KACC42780 genome (Park *et al.* 2014). Mapping of the KACC42780 *pheromone receptor* genes, *pheromone precursor* genes, and *pheromone receptor like* genes showed that the PR-a (*FvSTE3.1* and *FvPp1*) and the PR-b (*FvSTE3.2*, *FvPp2* and *FvPp3*) subloci were located on one chromosome. *Pheromone receptor like* genes *FvSTE3.s3* and *FvSTE3.s5* were located on the same chromosome as the PR subloci. *Pheromone receptor like* genes *FvSTE3.s1*, *FvSTE3.s2*, *FvSTE3.s4*, and *FvSTE3.s6* were located on different chromosomes. The distance between the PR-a and PR-b subloci on the chromosome was similar to that of the previously reported draft genome, 181 *vs.* 177 kb. The distance between the PR-a sublocus and *pheromone receptor like* gene *FvSTE3.s3* was found to be 43 kb less (138 *vs.* 181 kb). The distance between *pheromone receptor like* gene *FvSTE3.s3* and *FvSTE3.s5* was found to be much larger, at 304 kb compared to 184 kb (Figure 2, cf. Figure 1 in van Peer *et al.* 2011). The *FvSTE3.1* and *FvPp1* genes on the PR-a subloci of strain KACC4270 and L11 were arranged in the same way. Although some of the neighboring genes of the PR-a sublocus were arranged similarly, overall synteny of the PR-a sublocus region was not high.

The orientation of the *pheromone receptor* gene and the *pheromone precursor genes* on the PR-b sublocus of KACC42780 was found to be different from the reported draft genome. The *pheromone precursor* genes contained an outward instead of an inward orientation respective to the PR-b sublocus, and *pheromone receptor* gene *FvSTE3.2* was directed toward *FvPp3* instead of toward *FvPp2* (Figure 2, compare to Figure 1 in van Peer *et al.* 2011). This new arrangement of *pheromone receptor* genes and *pheromone precursor* genes on the PR-b sublocus of KACC42780 was similar to the arrangement of the *pheromone receptor* and *pheromone precursor* genes on the PR-b subloci of strain L11 and W23 (Figure 2C). Interestingly, strain L11 and strain KACC42780 contained the same PR-a sublocus, but different PR-b subloci. This indicated that the PR-a and PR-b subloci could recombine independently.

### Segregation analysis of HD and PR mating-type genes

Thirty one SSIs and F1 progeny of dikaryotic strain 1123 were examined to assess the occurrence of independent recombination between HD subloci and between PR subloci (Table S7). One of the 31 SSIs, no.24, showed a new combination of an HD-a and HD-b sublocus. SNPs in the *FvHd2-1* gene at the HD-a sublocus were specific for strain L11, while the HD-b sublocus contained the W23-specific *FvHd1-2* and *FvHd2-3* genes. To determine the site of recombination, seven different regions with SNPs specific for strain L11 or W23, located in between the HD-a and the HD-b subloci (Figure 1), were PCR amplified and sequenced. Specific SNPs for strain L11 were found in the PCR products of regions 1–6. Region seven contained specific SNPs for strain W23. Recombination therefore occurred between SNP-specific region six and seven. Unfortunately, sequence homology between strains L11 and W23 prevented further specification of the recombination site. It remained undetermined if recombination had occurred between the first and the second gene that flanked the HD-b sublocus, or within one of those genes.

Independent recombination of PR-a and PR-b was apparent from the genome data of strains L11 and KACC42780. However, no such recombination was observed in the 31 SSIs of strain 1123.

### Crossing of strains with different HD subloci

**Figure 5:**
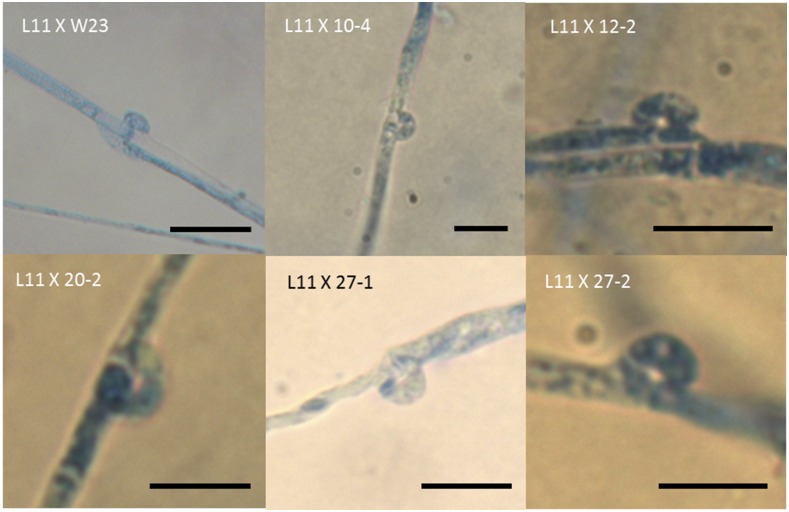
Microscopic images of clamp connections in *F. velutipes* colonies from compatible crosses between strain L11 with strains W23, 10-4, 12-2, 20-2, 27-1, and 27-2. The different strains that were mated with L11 generated different combinations of HD-b subloci. Bars in the pictures represent 10 µm.

Single spore isolate (SSI) no.24, which contained the HD-a sublocus of strain L11 and the HD-b sublocus of strain W23, was crossed with parental strain L11. This combined two identical HD-a subloci (SSI no.24 and L11 contained the *FvHd2-1* gene from L11) and two different HD-b subloci (SSI no.24 contained the *FvHd1-2* and *FvHd2-3* genes, and L11 contained the *FvHd1-3* and *FvHd2-4* genes, Figure 3). This new no.24/L11 dikaryon produced regular clamp connections, which demonstrated that the HD-b sublocus likely controled the HD pathway. Crossing of SSI no.24 and strain L22, which contained a different *FvHd2-1* gene (SNPs) at the HD-a sublocus, and the same *FvHd1-2* and *FvHd2-3* genes at the HD-b sublocus, did not result in colonies that formed proper clamps. Combinations of HD-a subloci with different copies (SNPs) of the *FvHd2-1* gene could not activate the HD pathway. Further crossing of strain L11 and a series of monokaryons with a *FvHd2-1* gene at the HD-a sublocus and different HD-b subloci confirmed that the HD pathway could be activated by multiple combinations of *FvHd1-3*/*FvHd2-4* (L11) with *FvHd1-2*/*FvHd2-3* (strains W23 and 12-2), *FvHd1-4*/*FvHd2-5* (strain 10-4, 27-1 and 27-2), and strain 20-2, of which the HD-b sublocus remained undetermined (Figure 5).

Poor conservation of the regions surrounding the PR-a and PR-b subloci had prevented the identification of PR subloci in addition to that of strain L11, W23, and KACC42780. Activity of the separate PR-a and PR-b subloci could therefore not be tested by specific crossings.

## Discussion

The identification of the first HD and PR mating type loci of *F. velutipes* in the draft genome of strain KACC42780, had indicated that the HD and PR mating-type loci consisted of two subloci, HD-a and HD-b and PR-a and PR-b, respectively (van Peer *et al.* 2011). In contrast to most mushroom-forming fungi, the HD-a and HD-b subloci of *F. velutipes* were found to be separated by 73 kb. This separation was explained by inversions in the genomic region that contained the HD locus. In addition, the *β-flanking* gene that is commonly associated with the HD locus, was not found near the HD-a or the HD-b subloci in *F. velutipes*. This particular organization of the *F. velutipes* HD subloci was supported by this study. A newer version of the *F. velutipes* genome of strain KACC42780 (Park *et al.* 2014), and the genomes of *F. velutipes* strain L11 and strain W23, all showed the same organization of the HD locus. It had remained unclear if the HD-a, the HD-b, or both HD subloci of *F. velutipes* were involved in mating. The segregation analysis in this study had shown that independent recombination is possible between the HD-a and HD-b subloci. If both HD subloci were active, new HD mating types could be generated this way.

The *FvHd2-1* gene of the HD-a sublocus had been reported to be specific for strain KACC42780 (*i.e.*, was not detected in a mating compatible strain), but no compatible HD-a sublocus was identified (van Peer *et al.* 2011). The present study found that the *FvHd2-1* gene of the HD-a sublocus was actually conserved in a large number of tested *F. velutipes* strains; however, not all *F. velutipes* strains contained the conserved *FvHd2-1* gene (Figure 3). Together with the previous report, where no *FvHd2-1* gene had been detected in strain KACC43777, the absence of the *FvHd2-1* gene at the HD-a subloci of multiple *F. velutipes* strains indicated that there is variation at this sublocus. To understand the regulation of the HD pathway in *F. velutipes*, it will be important to determine of what this variation consists. If no *FvHd* genes are present at other HD-a subloci, the HD-a sublocus might be degenerate. However, if only a single *FvHd1* gene (or a *FvHd1*/*FvHd2* gene pair) would be present, the HD-a sublocus might still be active. Functional HD subloci that contain a single *homeodomain* gene are not uncommon (for a review, see Kües 2015).

The data from a previous study concerning the HD-b subloci of strain KACC42780 and mating compatible strain KACC43777 were unclear. While the *FvHd1* gene on the HD-b sublocus of KACC42780 was also detected in strain KACC43777, the *FvHd2* gene on the HD-b sublocus of KACC42780 (*i.e.*, of the same functional pair), was not detected in strain KACC43777. This suggested a highly unlikely recombination within an *Hd1*/*Hd2* gene couple, which would complicate the function of HD-b (van Peer *et al.* 2011). In this study, we revealed a strong conservation over the largest parts (four-fifths) of the *FvHd1* and *FvHd2* genes. It was found that the PCR primers that had been used to detect the *FvHd1-1* gene in strain KACC42780 and KACC43777 bound within the conserved region of this gene. In contrast, the primers that were used to test for the presence of the *FvHd2-2* gene in strain KACC42780 and KACC43777, did not bind within the conserved region of *FvHd2-2*. This could well explain why the *FvHd1-1* primers generated highly similar sequences from strain KACC42780 and KACC4377, suggesting the presence of the same *FvHd1* gene, while the sequence for the *FvHd2-2* gene was specific for strain KACC42780. Most likely, therefore, is that the HD-b sublocus of strain KACC43777 contains a specific *Hd1*/*Hd2* gene couple, different from the *FvHd1* and *FvHd2* genes on the HD-b sublocus of strain KACC42780. Unfortunately we did not have strain KACC43777 or KACC42780 to test this hypothesis. The high conservation of the *FvHd* genes might challenge the importance of divergence in the non N-terminal parts of HD-encoding genes in their role of preventing recombination between alleles (*Hd1* genes or *Hd2* genes) on the same sublocus (Kües 2015, and references herein). Selection in favor of *Hd* genes encoding highly divergent C-terminus sequences seems to be absent (or failing) in *F. velutipes*. Yet, none of the new HD-b subloci (Figure 3) indicated separate recombination between *FvHd1* or *FvHd2* genes. In addition, no recombination of L11 *FvHd1* with W23 *FvHd2* genes had been observed in the HD-b subloci of the 31 SSIs that were used for the segregation analysis.

A total of four new types of HD-b subloci was identified in addition to the HD-b sublocus of KACC42780. Each of the, in total five, HD-b subloci contained a specific *Hd1*/*Hd2* gene couple (Figure 3). A function in activation of the HD pathway was derived for three of these subloci, using crosses between strain L11 and strains with similar HD-a, but different HD-b subloci (Figure 3 and Figure 5). Clearly, the HD-b sublocus is active.

The arrangement of the PR-a and PR-b subloci had been found to be unusual. The PR-a and PR-b subloci were separated by a very large, 177 kb genomic region. This large distance was supported (181 kb in this study) by the newest version of the genome sequence of strain KACC42780 (Park *et al.* 2014), as well as by the genomes of strain L11 and strain W23. Despite the large distance that separated the PR-a and the PR-b subloci, we did not observe independent recombination in a segregation population. However, the genomes of strain KACC42780 and strain L11 contained the same PR-a sublocus (Figure 2), while their PR-b subloci were different.

It had been deduced that the PR-a sublocus mediated activation of the PR pathway in a cross between mating compatible strains KACC42780 and KACC43777 (the same three *FvSTE3.2*, *FvPp2*, and *FvPp3* genes had been detected on the PR-b subloci of both strains; van Peer *et al.* 2011). Our data indicated that the PR-a subloci of the mating compatible strains L11 and W23 might be inactive in a cross between strain L11 and W23. Although the pheromone receptors at the PR-a subloci of L11 (FvSTE3.1) and W23 (FvSTE3.4) were clearly different, the predicted mature pheromones FvPP1 (L11) and FvPP4 (W23) would be identical (Figure S17). We might speculate that the mutation in FvPP1 could have inactivated this pheromone, preventing self-activation of the L11 PR-a sublocus, and allowing activation of pheromone receptor FvSTE3.1 by pheromone FvPP4. Possibly, recombination within the PR-a sublocus of L11 created an initially self-active locus with *FvSTE3.1* and *FvPp1*, followed by a mutation in FvPP1 that restored mating type specificity. Alternatively, *FvSTE3.1* might have recombined with *FvPp1* in the L11 PR-a sublocus after FvPP1 had lost its function. The possibility of within PR sublocus recombination is supported by studies on *C. cinerea* (Kües 2015, and references therein), and by the phylogenetic analysis that indicated that pheromone receptors FvSTE3.1 and FvSTE3.4 belong to different evolutionary clades (this study; James *et al.* 2004b, 2006; Riquelme *et al.* 2005; Kües *et al.* 2011).

The PR-b subloci of strains L11, W23, and KACC42780 each contained a unique *pheromone receptor* gene and two unique *pheromone precursor* genes (Figure 2). Crosses between strain L11 and W23 (which are known to generate proper dikaryons) would introduce two nonself pheromones to the pheromone receptor of strain L11, and two nonself pheromones to the pheromone receptor of W23. It is highly likely that at least one of these four combinations would be compatible, and thus that the PR-b sublocus is active.

Based on this study, we conclude that *F. velutipes* has two HD subloci. The HD-b sublocus is active, while the HD-a sublocus might be active and requires additional analysis. We further conclude that the PR locus consists of two subloci. The PR-a sublocus was previously confirmed to be active, and our data strongly indicate that the PR-b sublocus is active. Both the HD and the PR subloci have been found to be able to recombine, and new mating types could be generated. We are currently using transformation assays to confirm the function of individual mating-type genes, and continue to identify additional HD and PR subloci. This should help to establish if all four MAT subloci can be active, and which particular MAT genes can interact.

## 

## Supplementary Material

Supplemental Material
