# Supplementary material for: Advances in Understanding Mating Type Gene Organization in the Mushroom-Forming Fungus Flammulina velutipes
Source: G3 (Bethesda). 2016 Sep 9;6(11):3635–45. doi: 10.1534/g3.116.034637 (PMC5100862; doi:10.1534/g3.116.034637)
Supplement: Supplemental Material [file supp_6_11_3635__index.html]

Advances in Understanding Mating Type Gene Organization in the Mushroom Forming Fungus Flammulina velutipes — Advances in Understanding Mating Type Gene Organization in the Mushroom-Forming Fungus Flammulina velutipes — Supplemental Material 

# Advances in Understanding Mating Type Gene Organization in the Mushroom-Forming Fungus *Flammulina velutipes*

## Supplemental Material for Wang, *et al*, 2016

**Files in this Data Supplement:**

- Table S1 - Strains used in this study. (.pdf, 199 KB)
- Table S2 - Primers used in this study. (.pdf, 240 KB)
- Table S3 - GenBank accession numbers of the *F. velutipes* mating type genes. (.pdf, 285 KB)
- Table S4 - Parameters of the genome sequences of strain W23 and L11. (.pdf, 198 KB)
- Table S5 - Parameters of the predicted genes in the genomes of W23 and L11. (.pdf, 198 KB)
- Table S6 - Positions and lengths of the predicted transmembrane domains in the pheromone receptors of *F. velutipes*. (.pdf, 242 KB)
- Table S7 - Segregation analysis of HD and PR subloci in single spore isolates (SSIs) of a L11ï¿½W23 dikaryon. (.pdf, 202 KB)
- Figure S1 - Alignment of the HD-a subloci from the genomes of strain L11, W23, KACC42780 and additionally cloned HD-a subloci. (.tif, 1858 KB)
- Figure S2 - Alignment of the HD2 protein sequences from the HD-a and HD-b subloci of different *F. velutipes* strains. (.tif, 4,465 KB)
- Figure S3 - Visual representation of DNA sequence similarity between the genes on the five types of HD-b subloci. (.tif, 702 KB)
- Figure S4 - Alignment of the *FvHd1* genes on the HD-b subloci of *F. velutipes*. (.gif, 1,832 KB)
- Figure S5 - Alignment of the *FvHd2* genes on the HD-b subloci of *F. velutipes*. (.tif, 1,472 KB)
- Figure S6 - Alignment of the HD1 protein sequences from the HD-b subloci of different *F. velutipes* strains. (.tif, 2,971 KB)
- Figure S7 - Alignment of the HD2 protein sequences from the HD-b subloci of different *F. velutipes* strains. (.tif, 3,946 KB)
- Figure S8 - Alignment of the HD1 protein sequences from the HD-b subloci of different *F. velutipes* strains, including the HD1 proteins of which only partial sequences had been obtained. (.tif, 293 KB)
- Figure S9 - Alignment of the HD2 protein sequences from the HD-b subloci of different *F. velutipes* strains, including the HD2 proteins of which only partial sequences had been obtained. (.tif, 436 KB)
- Figure S10 - Alignment of the HD1 proteins that correspond to a specific HD sublocus in *L. edodes*. (.tif, 387 KB)
- Figure S11 - Alignment of the HD2 proteins that correspond to a specific HD sublocus in *L. edodes*. (.tif, 552 KB)
- Figure S12 - (A) Tree diagram indicating the relative distance between the aligned HD1 proteins (alpha) of *S. commune*. (B) Alignment of the HD1 proteins (alpha) of *S. commune*. (.jpg, 5,103 KB)
- Figure S13 - (A) Tree diagram indicating the relative distance between the aligned HD1 proteins (beta) of *S. commune*. (B) Alignment of the HD1 proteins (beta) of *S. commune*. (.jpeg, 4,853 KB)
- Figure S14 - (A) Tree diagram indicating the relative distance between the aligned HD2 proteins of *S. commune*. (B) Alignment of the HD2 proteins of *S. commune*. (.gif, 4,627 KB)
- Figure S15 - (A) Tree diagram indicating the relative distance between the aligned HD1 proteins of the b1 locus of *C. cinerea*. (B) Alignment of the HD1 proteins of the b1 locus of *C. cinerea*. (.tif, 4,705 KB)
- Figure S16 - (A) Tree diagram indicating the relative distance between the aligned HD2 proteins of *C. cinerea*. (B) Alignment of the HD2 proteins of *C. cinerea*. (.jpg, 4,448 KB)
- Figure S17 - Alignment of the pheromone precursors of *F. velutipes*. (.tif, 341 KB)
- Figure S18 - Analyses confirming the additional tryptophan (W) in FvPP1 of strain L11. (.tif, 2,677 KB)
