# Supplementary material for: Advances in Understanding Mating Type Gene Organization in the Mushroom-Forming Fungus Flammulina velutipes
Source: G3 (Bethesda). 2016 Sep 9;6(11):3635–45. doi: 10.1534/g3.116.034637 (PMC5100862; doi:10.1534/g3.116.034637)
Supplement: Supplemental Material [file supp_g3.116.034637_TableS1.pdf]

Table S1 Strains used in this study

Strain	Source	Single spore no.
F0010	Fungal Station, Agriculture Office of Fujian Province, China	10-1
		10-3
		10-4
F0012	Huangzhong Agriculture University, Wuhan, Hubei Province, China	12-1
		12-2
		12-3
F0020	Edible Fungi Center of Sichuan Academy of Agricultural Sciences, Sichuan Province, China	20-1
		20-2
		20-3
F0025	Sanming Mycological Institute of Fujian Province, China	25-1
		25-3
		25-4
F0027	Sanming Mycological Institute of Fujian Province, China	27-1
		27-2
		27-3
FL19	Fujian Edible Fungi Germplasm Resource Collection Center of China	L11
FL8801	Fujian Edible Fungi Germplasm Resource Collection Center of China	W23
KACC42780	Mushroom Research Division, National Institute of Horticultural and Herbal Science, Rural Development Administration, Suwon, Republic of Korea	
