# Supplementary material for: Advances in Understanding Mating Type Gene Organization in the Mushroom-Forming Fungus Flammulina velutipes
Source: G3 (Bethesda). 2016 Sep 9;6(11):3635–45. doi: 10.1534/g3.116.034637 (PMC5100862; doi:10.1534/g3.116.034637)
Supplement: Supplemental Material [file supp_g3.116.034637_TableS2.pdf]

Table S2 Primers used in this study

Primer name	Sequence (5'-3')
A)	
matAa-F	TCAGGTGGAAGTTTGGGTA
matAa-R	GGGCAATGCGTAGGGTAT
matAb-1F	TGACACGCCTGGTCTCCTG
matAb-1R	GCGAGTGCCCTCTGCTACAT
matAb-2F	ACGCAGCACGCTTGAGAA
matAb-2R	GGAAGATGGTGGCGAAGA
L11Ste3.1F	GTGTTTCGGTCTGCCTGTC
L11Ste3.1R	GTGGTGGTCGTGGGTGGTT
L11Ste3.5F	AGTGTCGTCCCAAGAAATCC
L11Ste3.5R	TTAGTCCCGTCTGTCTAAAGGTAT
W23Ste3.3F	GGCGAGCAGAAGGACGAAC
W23Ste3.3R	CCCGCATCCATTCTATGTATCA
W23Ste3.4F	GTGGCTGAGTATGGTGGTTT
W23Ste3.4R	GGCTGCTATCCTGCTCTGTA
B)	
P1F	GCGTGGCATTATCCTCTAC
P1R	GCGACAGCGACACCTTACT
P2F	AAACGCCAATATCGTAGAAC
P2R	CTCAAAGAGCCTTACCTCAA
P3F	GCAAGCACGAAACCTCAG
P3R	GGGAATCGGTCACGCAAT
P4F	ATTCGGAAACGACAGTAGCA
P4R	ACGGGACGATTTAGTGGG
P5F	TACTCTGGCGTCTTTGGAT
P5R	TTACGTGCTTCGTGACTGATA
P6F	GAACCACGCTAACATGAGACC
P6R	CGTAGTGAGCCCAACCAAA
P7F	TTTCAAGGAGCATTCGTTAC
P7R	TATCCCTTAGCATCAACAC
C)	
matAaLong-F	CCCACGCTCCTCGCTGTAA
matAaLong-R	ACGCAGTTCGGGCACCTCT
matAbLong-F	CGAGGGAAAGCATAGTAGGAG
matAbLong-R	AGCGGTGATTAGTAGTTGTAGTTG
D)	
L11-pp1F	CTAACATTTTTCCAACCG
L11-pp1R	AACATTCTGCCTTCCCGA

(A) Primers used for segregation analysis. (B) Primers used for identification of the exchange site of the HD-a and HD-b subloci. (C) Primers used for cloning of the HD-a and the HD-b subloci in additional *F. velutipes* strains. (D) Primers used for cloning and resequencing of the *FvPp1* gene of strain L11.
