# Supplementary material for: Advances in Understanding Mating Type Gene Organization in the Mushroom-Forming Fungus Flammulina velutipes
Source: G3 (Bethesda). 2016 Sep 9;6(11):3635–45. doi: 10.1534/g3.116.034637 (PMC5100862; doi:10.1534/g3.116.034637)
Supplement: Supplemental Material [file supp_g3.116.034637_TableS3.pdf]

Table S3 GenBank accession numbers of the *F. velutipes* mating type genes

Gene name	GenBank accession number	Gene name	GenBank accession number
Hd1-1/Hd2-2^{KACC42780}	HQ630588.1	*STE3.s5*^{W23}	KC208610.1
Hd2-1/MIP^{KACC42780}	HQ630589.1	*STE3.s6*^{W23}	KT808674.1
Pp1^{KACC42780}	HQ630597.1	*Hd1-3/Hd2-4*^{L11}	KC208594.1
STE3.1^{KACC42780}	HQ630590.1	*Hd2-1/MIP*^{L11}	KC208595.2
Pp2/STE3.2/Pp3^{KACC42780}	HQ630591.1	*Pp1*^{L11}	KC208612.1
STE3.s1^{KACC42780}	HQ630592.1	*STE3.1*^{L11}	KC208596.1
STE3.s2^{KACC42780}	HQ630593.1	*Pp8/STE3.5/Pp7*^{L11}	KC208597.1
STE3.s3^{KACC42780}	HQ630594.1	*STE3.s1*^{L11}	KC208598.2
STE3.s4^{KACC42780}	HQ630595.1	*STE3.s2*^{L11}	KC208599.1
STE3.s5^{KACC42780}	HQ630596.1	*STE3.s3*^{L11}	KC208600.2
STE3.s6^{KACC42780}	BK009409.1	*STE3.s4*^{L11}	KC208601.2
Hd1-2/Hd2-3^{W23}	KC208604.1	*STE3.s5*^{L11}	KC208602.1
Hd2-1/MIP^{W23}	KC208603.2	*STE3.s6*^{L11}	KT808675.1
Pp5/STE3.3/Pp6^{W23}	KC208605.1	*Hd1-4/Hd2-5*$^{27-1}$	KT808673.1
Pp4/STE3.4^{W23}	KC208611.1	*Hd1-5/Hd2-6*$^{25-1}$	KT808672.1
STE3.s1^{W23}	KC208606.2	*Hd1-6*$^{27-3}$	KT808676.1
STE3.s2^{W23}	KC208607.1	*Hd2-7*$^{27-3}$	KT808677.1
STE3.s3^{W23}	KC208608.2	*Hd2-8*$^{20-2}$	KT808678.1
STE3.s4^{W23}	KC208609.2	*Hd2-9*$^{25-4}$	KT808679.1

The corresponding strains of each gene are indicated by superscript.
