# Supplementary material for: Advances in Understanding Mating Type Gene Organization in the Mushroom-Forming Fungus Flammulina velutipes
Source: G3 (Bethesda). 2016 Sep 9;6(11):3635–45. doi: 10.1534/g3.116.034637 (PMC5100862; doi:10.1534/g3.116.034637)
Supplement: Supplemental Material [file supp_g3.116.034637_TableS4.pdf]

Table S4 Parameters of the genome sequences of strain W23 and L11

Parameter	Scaffold		Contig	
	W23	L11	W23	L11
Total Num(#)	1,866	1,858	42,049	28,590
Total Length(bp)	35,131,566	34,753,824	33,786,700	34,561,456
N50(bp)	72,366	66,831	1,547	2,970
N90(bp)	7,021	6,522	345	549
Max length(bp)	519,350	581,968	16,175	22,173
Average Length(bp)	18,827	18,704	803	1,208
