# Supplementary material for: Advances in Understanding Mating Type Gene Organization in the Mushroom-Forming Fungus Flammulina velutipes
Source: G3 (Bethesda). 2016 Sep 9;6(11):3635–45. doi: 10.1534/g3.116.034637 (PMC5100862; doi:10.1534/g3.116.034637)
Supplement: Supplemental Material [file supp_g3.116.034637_TableS5.pdf]

Table S5 Parameters of the predicted genes in the genomes of W23 and L11

Parameter	W23	L11
Gene Number(#)	11,071	11,526
Total Gene Length (bp)	20,522,473	21,487,725
Total Coding Sequence Length (bp)	15,802,023	16,986,513
Average Gene Length (bp)	1,854	1,864
Average Coding Sequence Length (bp)	1,428	1473
Gene / Genome	58.42%	61.83%
Coding Sequences/Genome	44.98%	48.88%
Max Gene Length (bp)	18,130	17,562
Min Gene Length (bp)	92	92
