# Supplementary material for: Advances in Understanding Mating Type Gene Organization in the Mushroom-Forming Fungus Flammulina velutipes
Source: G3 (Bethesda). 2016 Sep 9;6(11):3635–45. doi: 10.1534/g3.116.034637 (PMC5100862; doi:10.1534/g3.116.034637)
Supplement: Supplemental Material [file supp_g3.116.034637_TableS6.pdf]

Table S6 Positions and lengths of the predicted transmembrane domains in the pheromone receptors of *F. velutipes*

Protein	Protein	TM1	TM2	TM3	TM4	TM5	TM6	TM7
STE3.1^{KACC42780}	447	18/5-22	23/29-51	19/71-89	23/110-132	23/147-169	23/199-221	23/258-280
STE3.2^{KACC42780}	505	23/4-26	23/38-60	18/70-87	23/110-132	23/160-182	23/203-225	20/268-287
STE3.3^{W23}	508	23/5-27	23/34-56	23/71-93	23/113-135	23/163-185	23/206-228	23/267-289
STE3.4^{W23}	391	20/10-29	20/36-55	20/70-89	23/110-132	23/161-183	23/204-226	20/271-290
STE3.5^{L11}	501	23/5-27	23/34-56	23/71-93	23/113-135	23/158-180	23/217-239	20/271-290
STE3.1^{L11}	457	18/5-22	23/29-51	23/66-88	23/109-131	23/151-173	23/204-226	23/263-285

Length of proteins and transmembrane (TM) domains are given in amino acids. TM positions are indicated by the size of the TM domain and the respective amino acid numbers as counted from the N-terminus towards the C-terminus. **Example:** TM1, (18/5-22) means that the first transmembrane domain (of STE3.1) is 18 amino acids long and runs from amino acid number 5 to 22.
