# Supplementary material for: Advances in Understanding Mating Type Gene Organization in the Mushroom-Forming Fungus Flammulina velutipes
Source: G3 (Bethesda). 2016 Sep 9;6(11):3635–45. doi: 10.1534/g3.116.034637 (PMC5100862; doi:10.1534/g3.116.034637)
Supplement: Supplemental Material [file supp_g3.116.034637_TableS7.pdf]

Table S7 Segregation analysis of HD and PR subloci in single spore isolates (SSIs) of a L11×W23 dikaryon

SSI	HD		PR	
	HD-a	HD-b	PR-a	PR-b
no.1	L	L	W	W
no.2	L	L	L	L
no.3	L	L	L	L
no.5	L	L	L	L
no.10	L	L	L	L
no.15	W	W	W	W
no.22	W	W	W	W
no.23	W	W	W	W
**no.24**	L	W	W	W
no.25	W	W	L	L
no.28	W	W	L	L
no.29	W	W	W	W
no.33	W	W	W	W
no.34	L	L	W	W
no.36	W	W	L	L
no.38	W	W	L	L
no.39	W	W	L	L
no.41	W	W	L	L
no.42	W	W	W	W
no.43	W	W	L	L
no.44	W	W	W	W
no.45	L	L	L	L
no.46	W	W	L	L
no.47	L	L	W	W
no.48	W	W	W	W
no.49	W	W	L	L
no.50	W	W	L	L
no.51	W	W	L	L
no.53	W	W	W	W
no.54	W	W	L	L
no.55	W	W	W	W

The origins of subloci as determined by detection of L11 (L) or W23 (W) specific single nucleotide polymorphisms.
