# Supplementary figures and images for: Advances in Understanding Mating Type Gene Organization in the Mushroom-Forming Fungus Flammulina velutipes
Source: G3 (Bethesda). 2016 Sep 9;6(11):3635–45. doi: 10.1534/g3.116.034637 (PMC5100862; doi:10.1534/g3.116.034637)

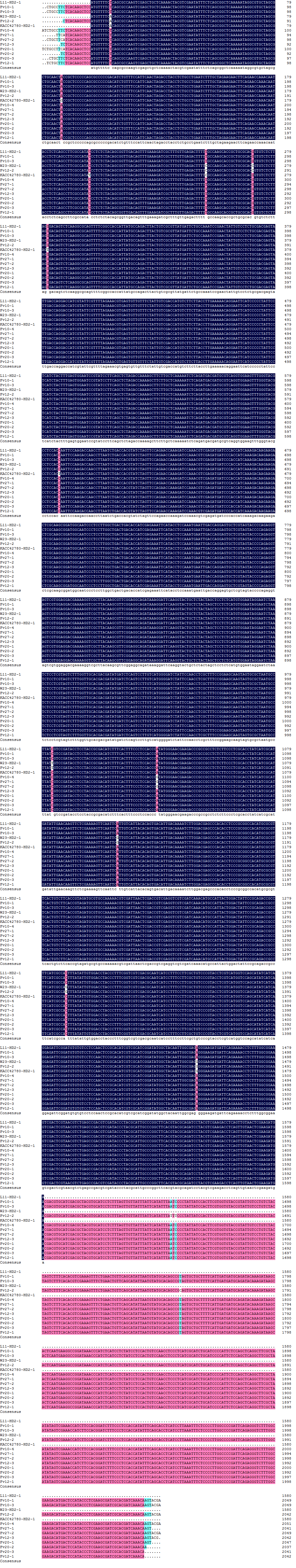

Supplement: Supplemental Material [file supp_g3.116.034637_FigureS1.tif]

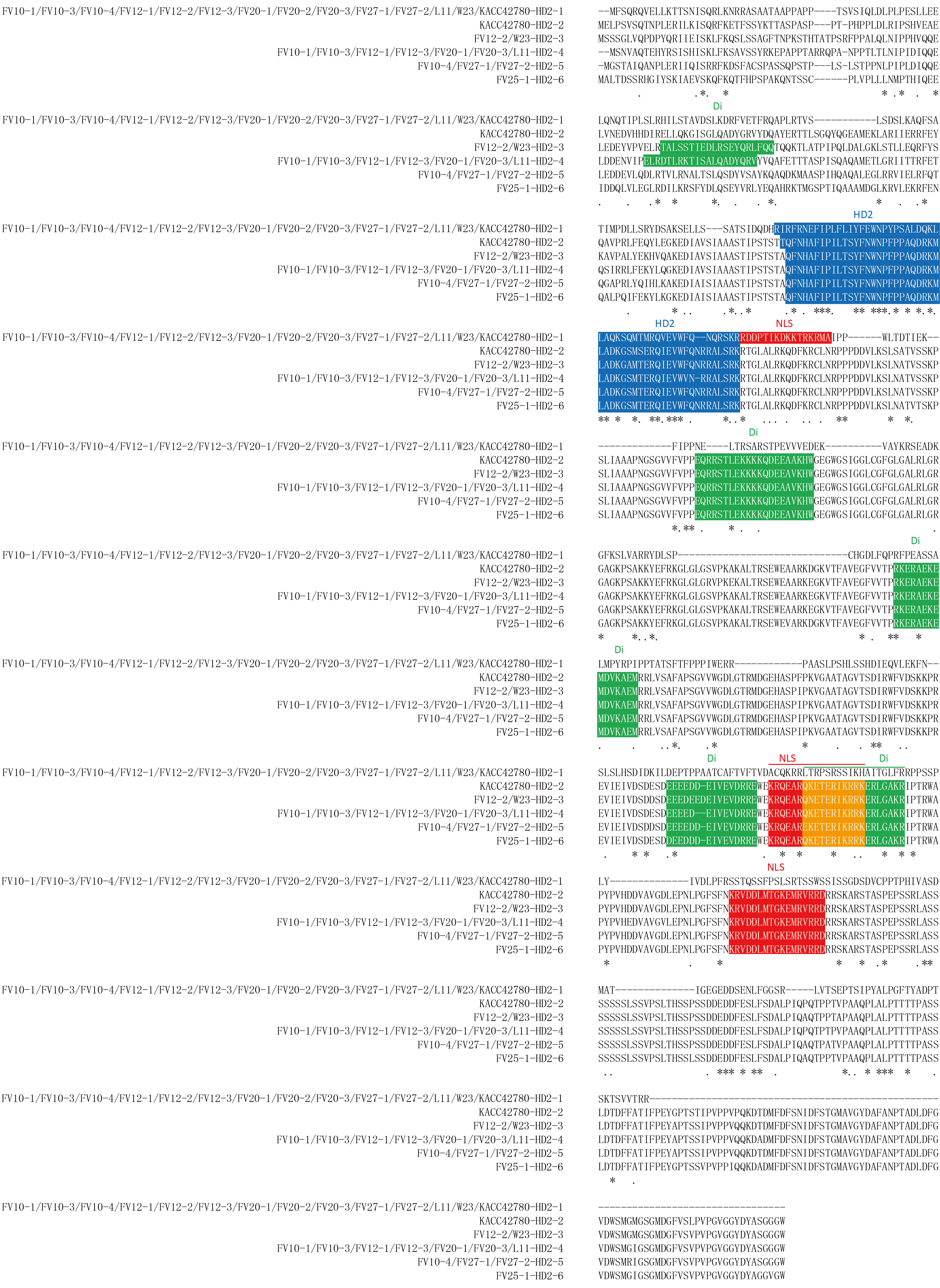

Supplement: Supplemental Material [file supp_g3.116.034637_FigureS2.tif]

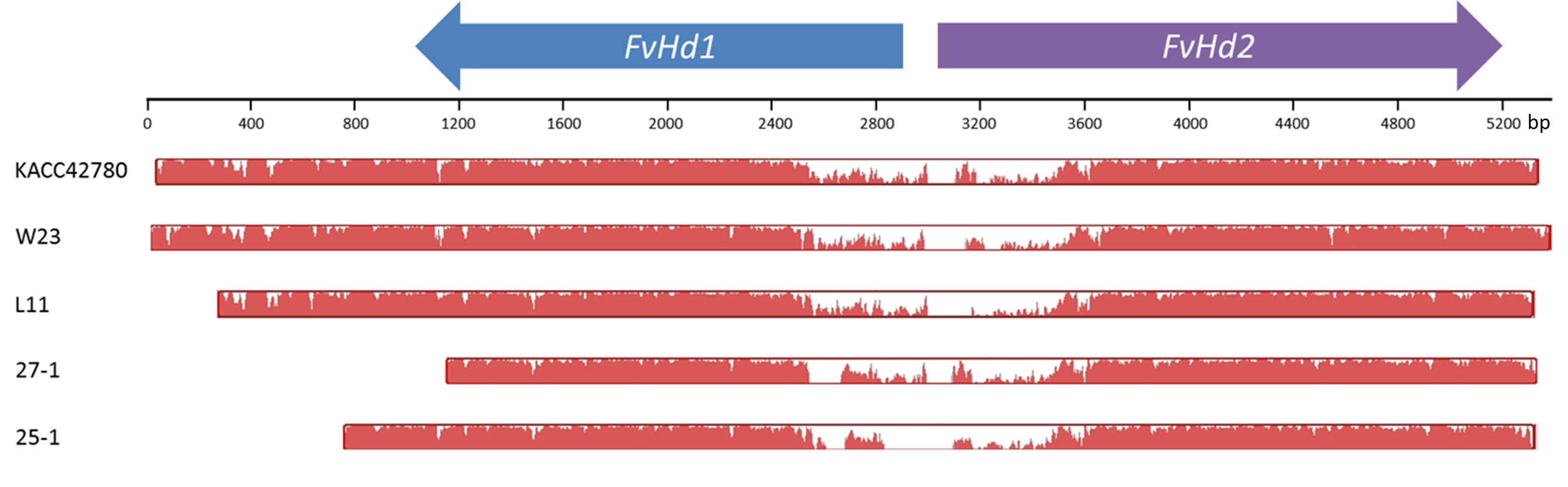

Supplement: Supplemental Material [file supp_g3.116.034637_FigureS3.tif]

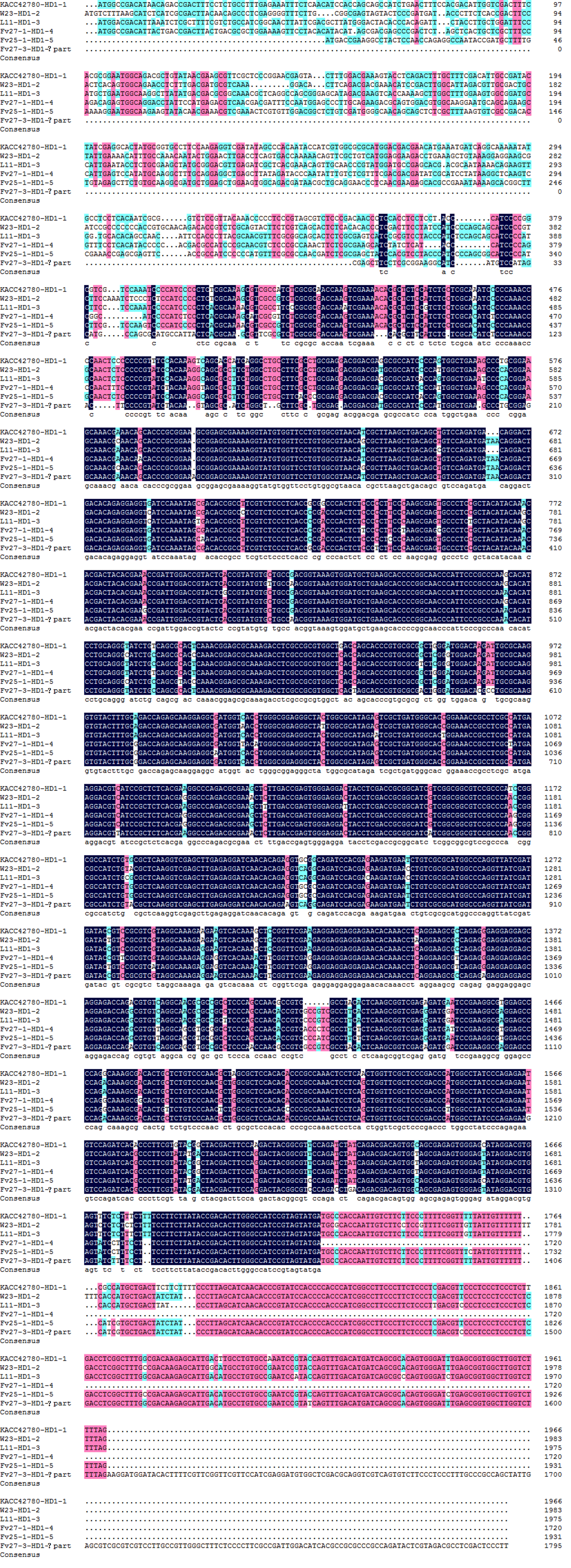

Supplement: Supplemental Material [file supp_g3.116.034637_FigureS4.gif]

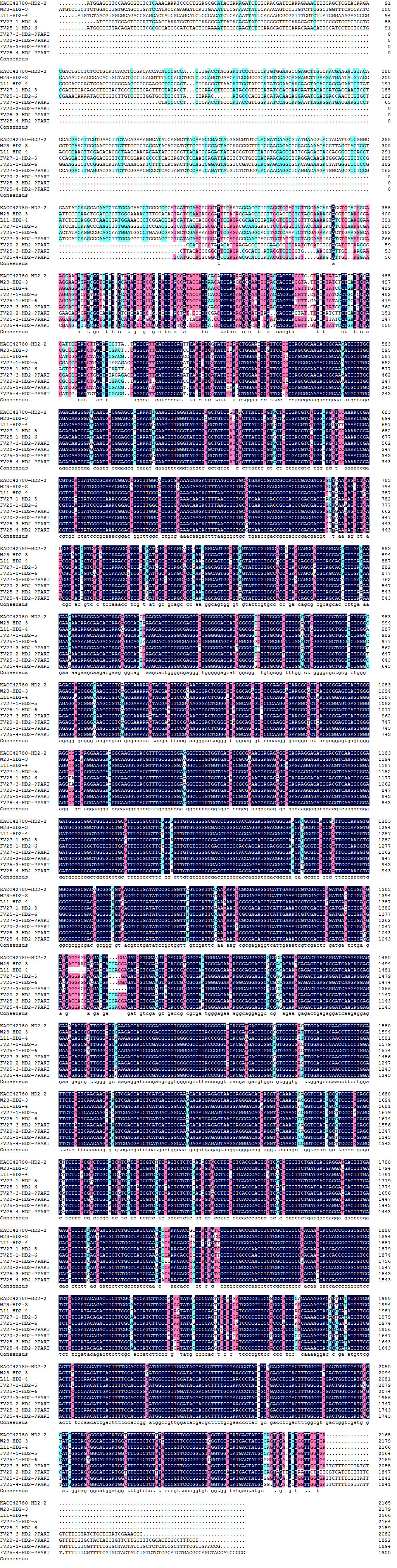

Supplement: Supplemental Material [file supp_g3.116.034637_FigureS5.tif]

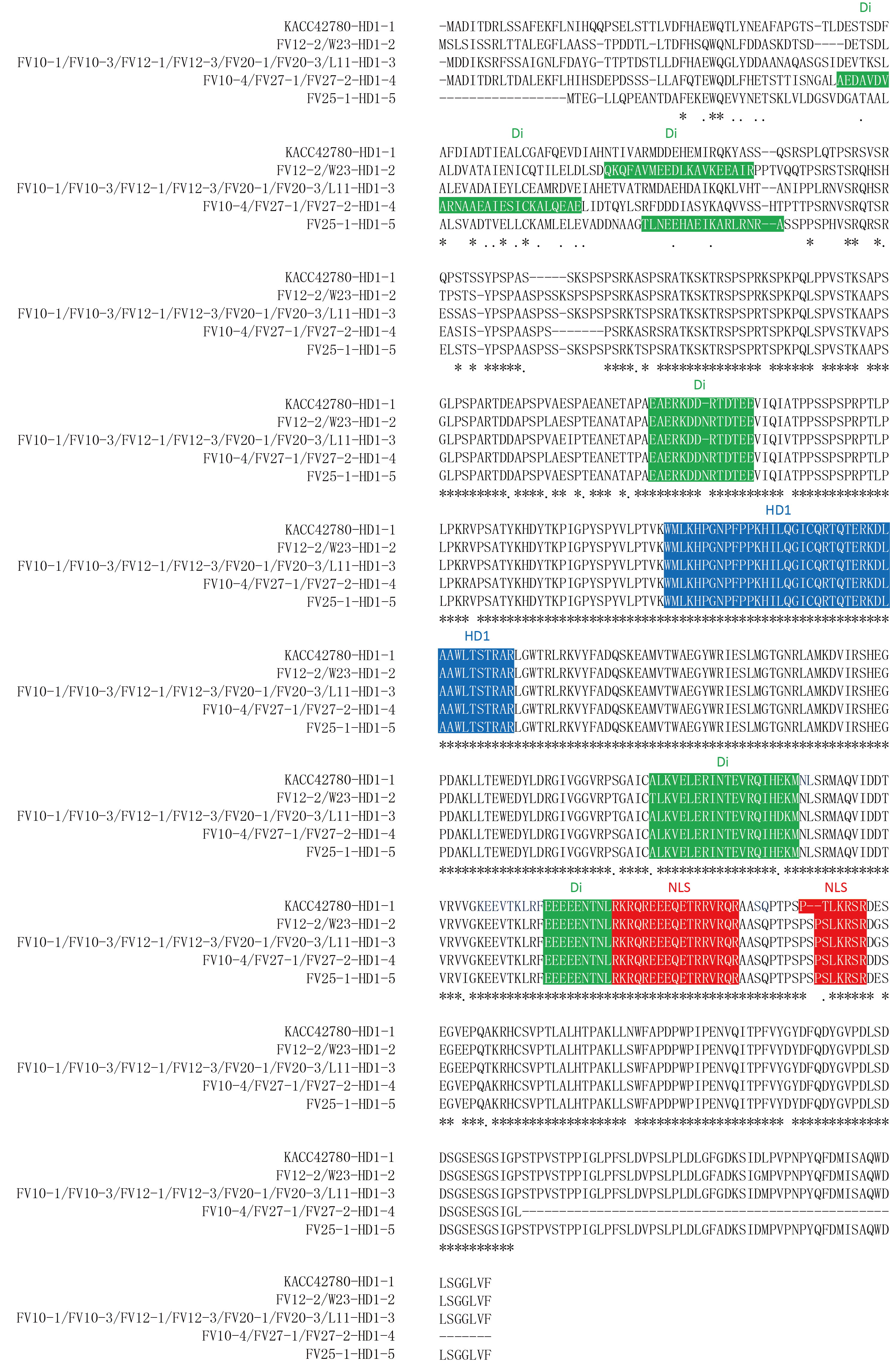

Supplement: Supplemental Material [file supp_g3.116.034637_FigureS6.tif]

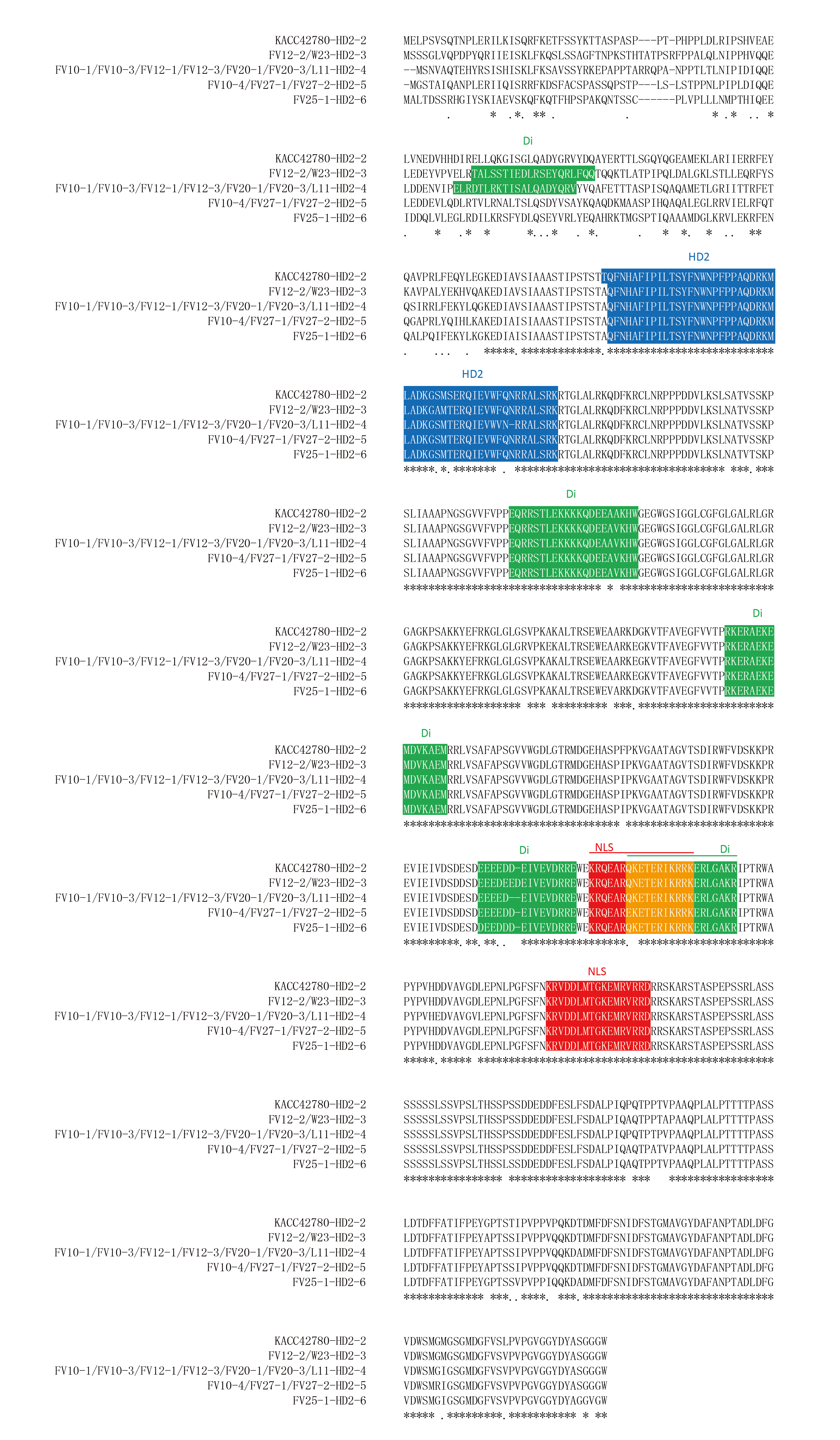

Supplement: Supplemental Material [file supp_g3.116.034637_FigureS7.tif]

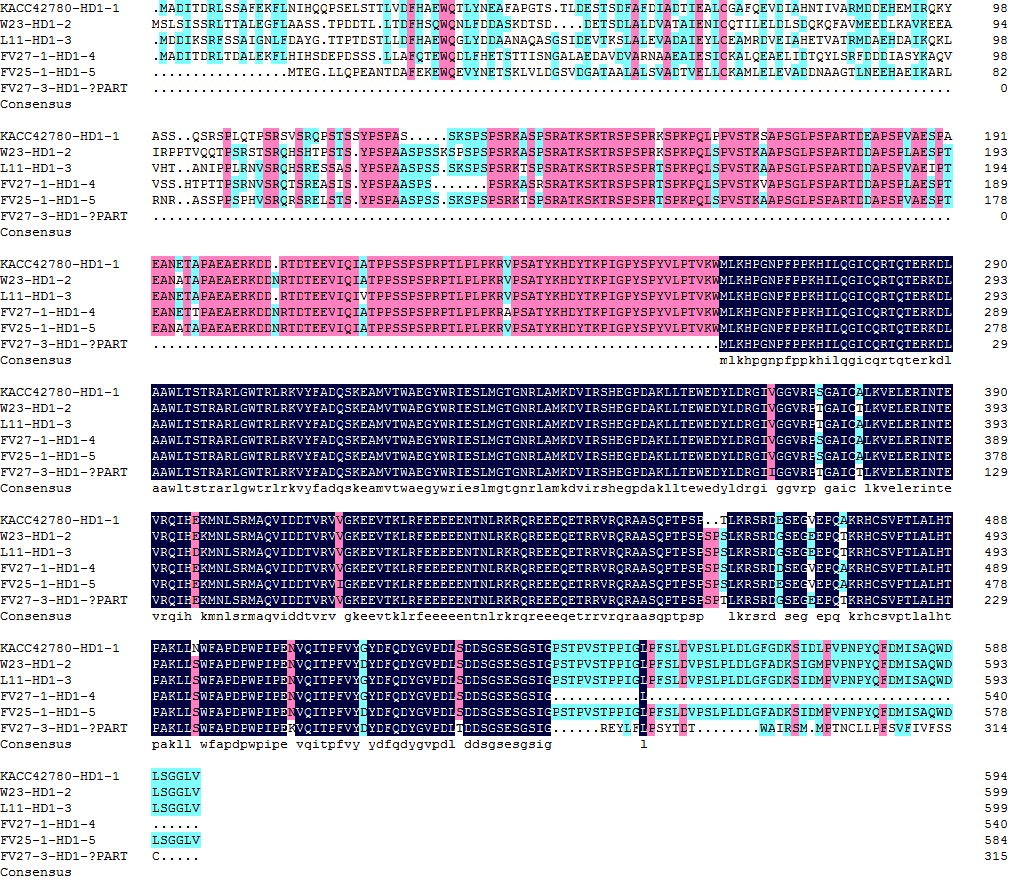

Supplement: Supplemental Material [file supp_g3.116.034637_FigureS8.tif]

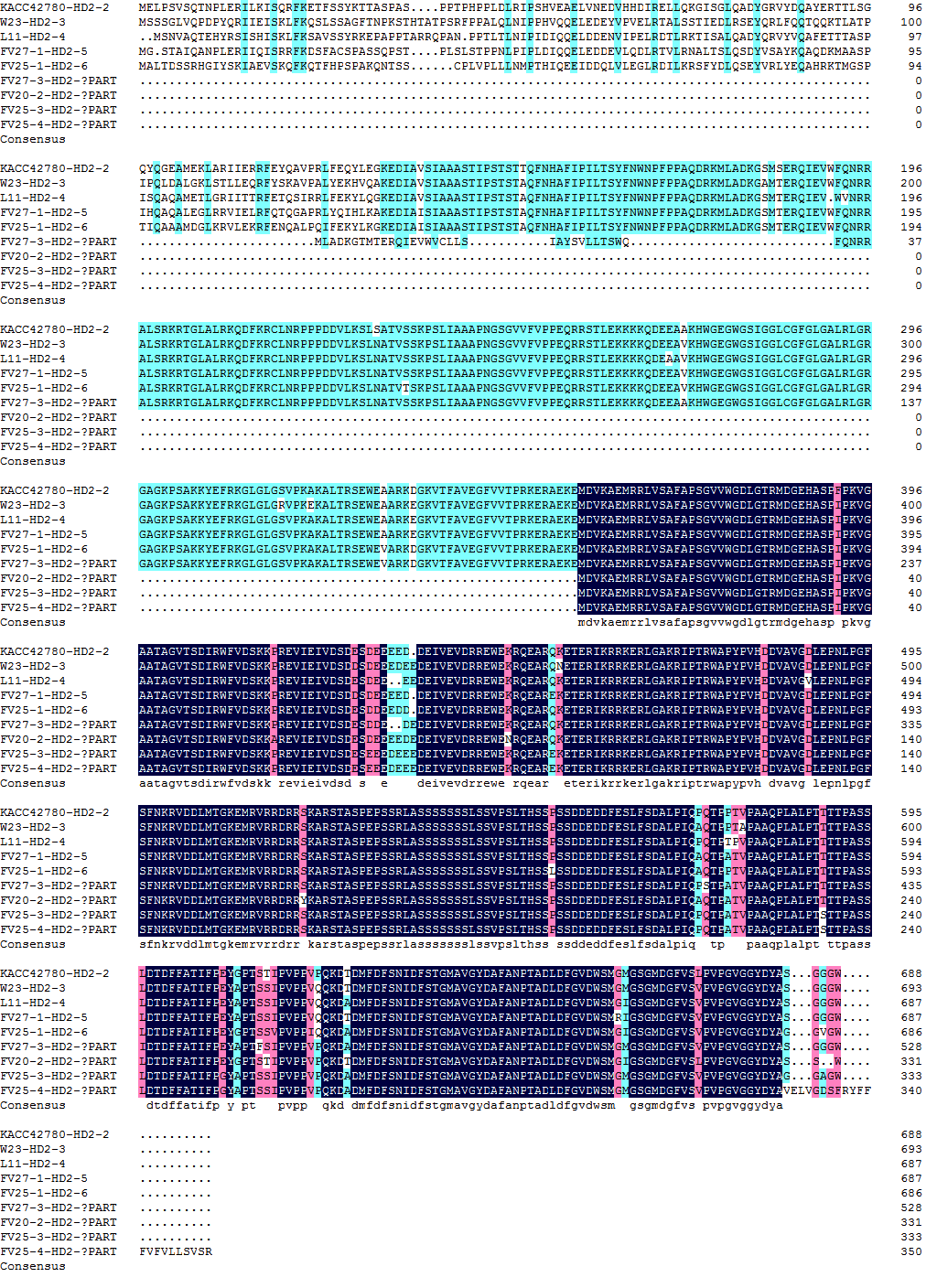

Supplement: Supplemental Material [file supp_g3.116.034637_FigureS9.tif]

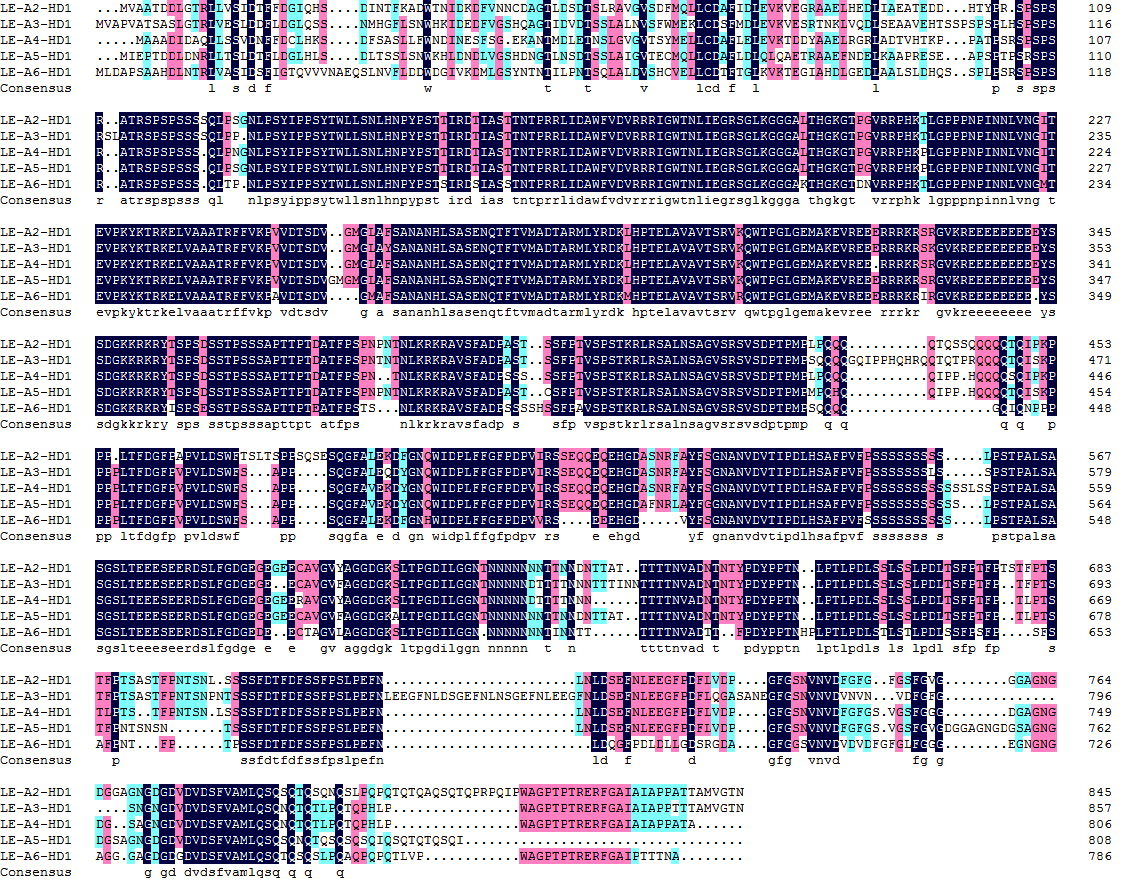

Supplement: Supplemental Material [file supp_g3.116.034637_FigureS10.tif]

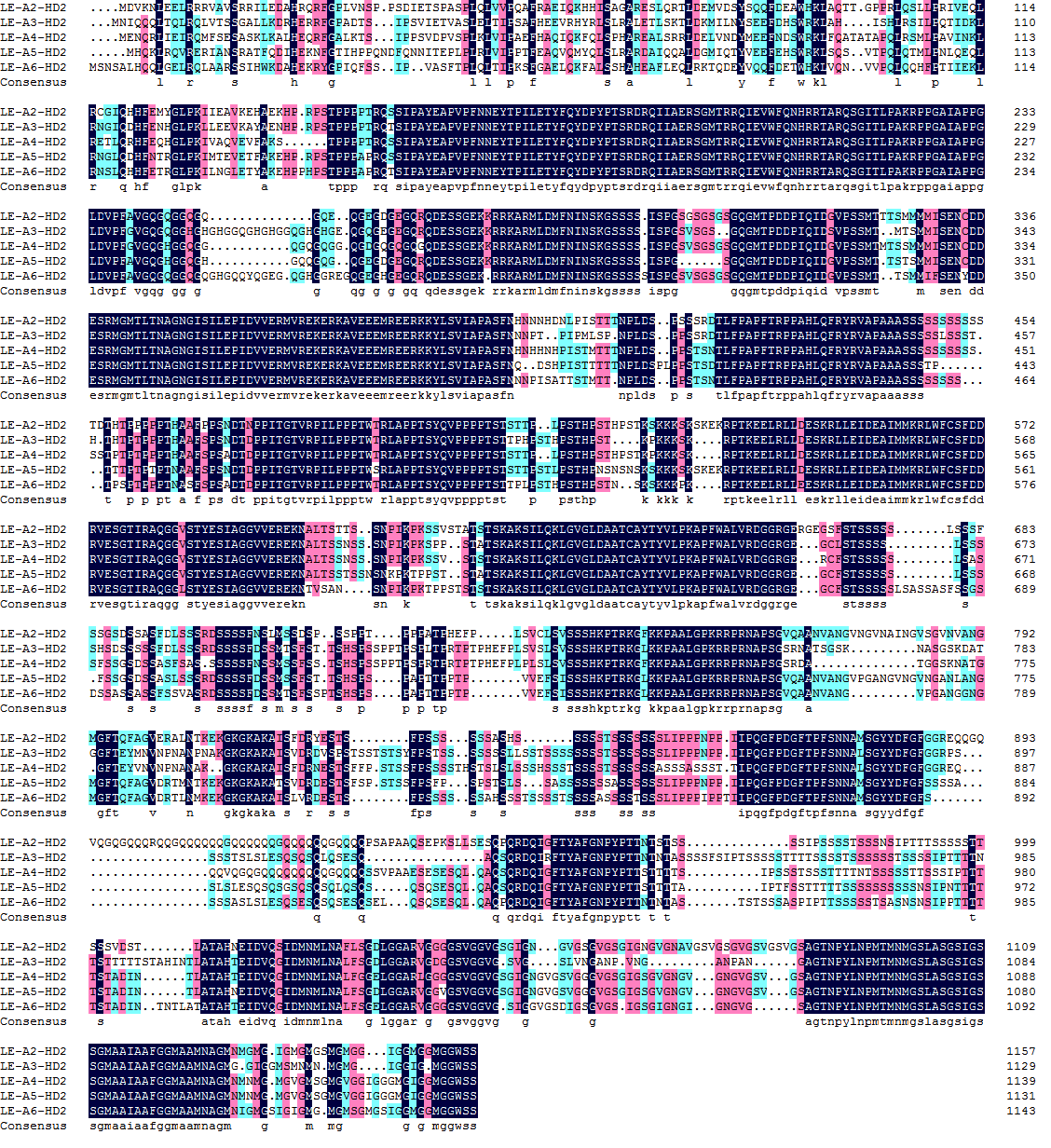

Supplement: Supplemental Material [file supp_g3.116.034637_FigureS11.tif]

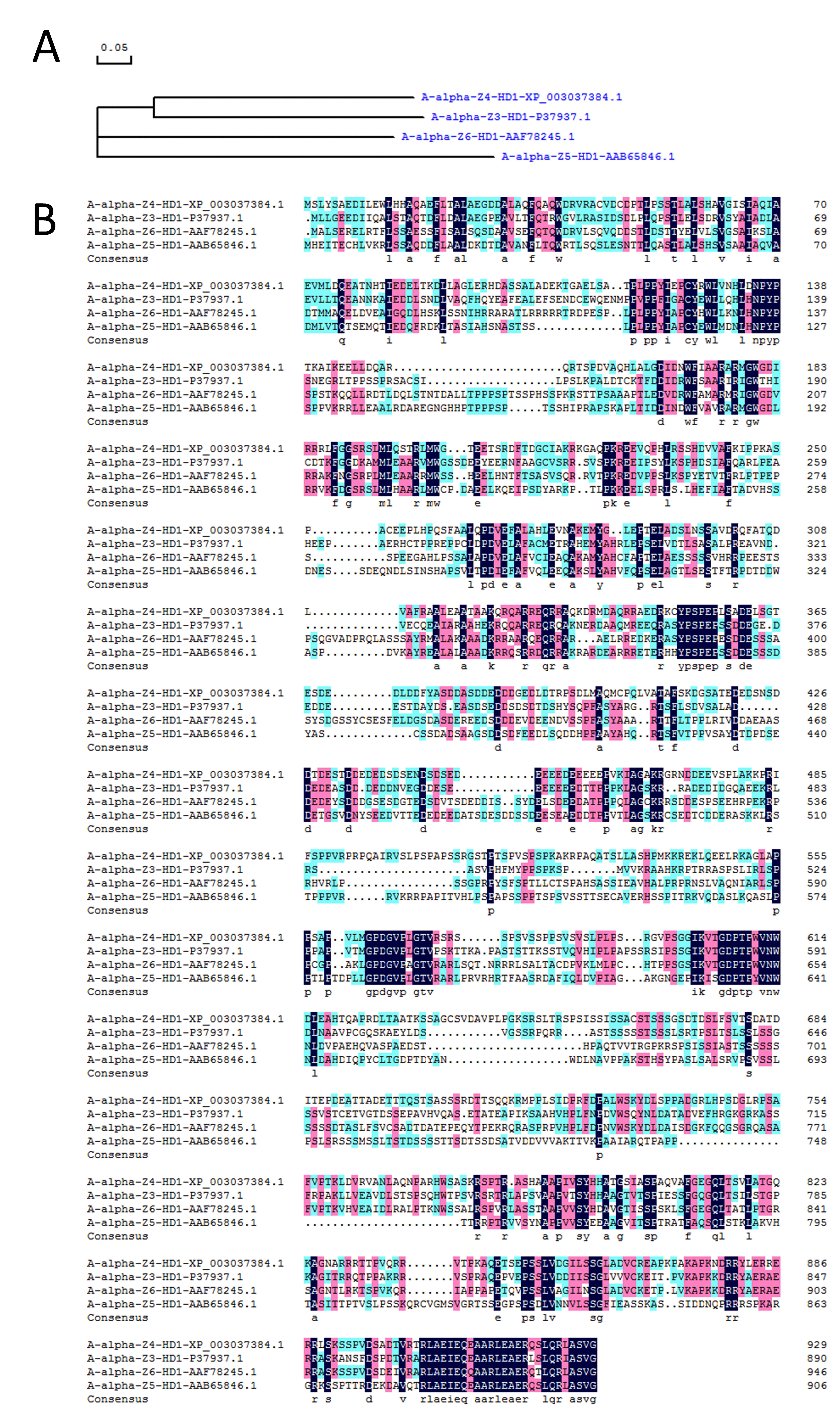

Supplement: Supplemental Material [file supp_g3.116.034637_FigureS12.jpg]

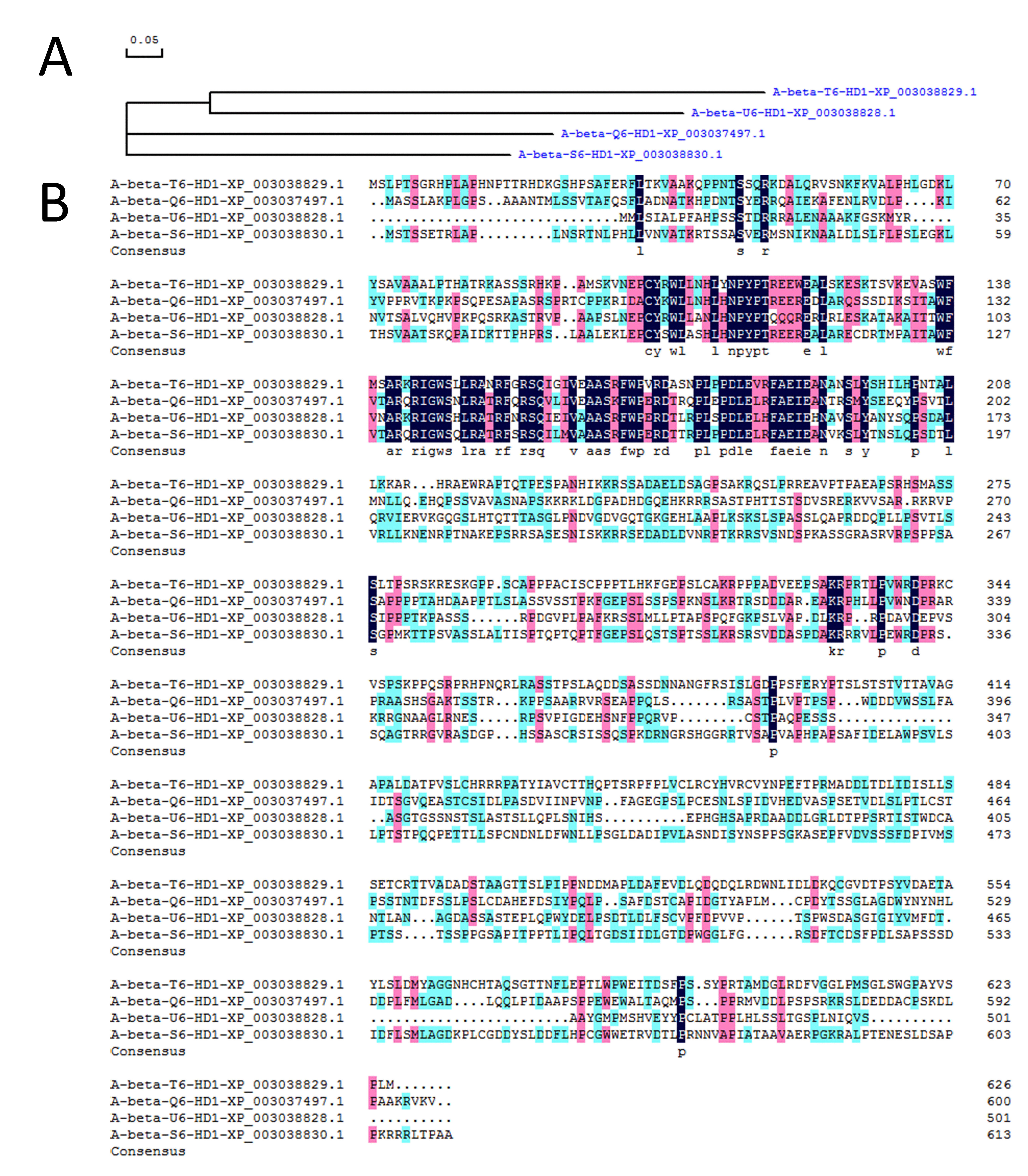

Supplement: Supplemental Material [file supp_g3.116.034637_FigureS13.jpg]

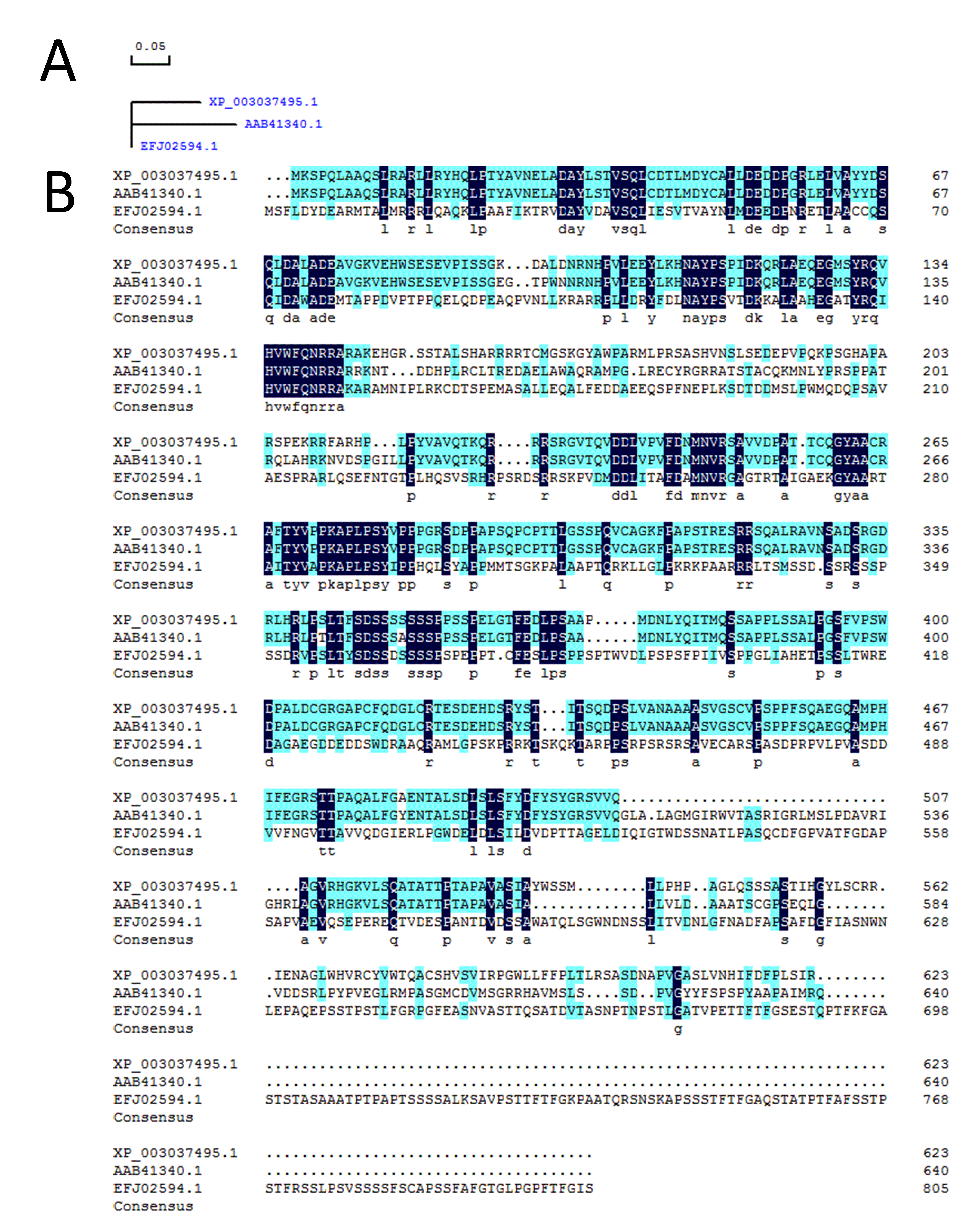

Supplement: Supplemental Material [file supp_g3.116.034637_FigureS14.gif]

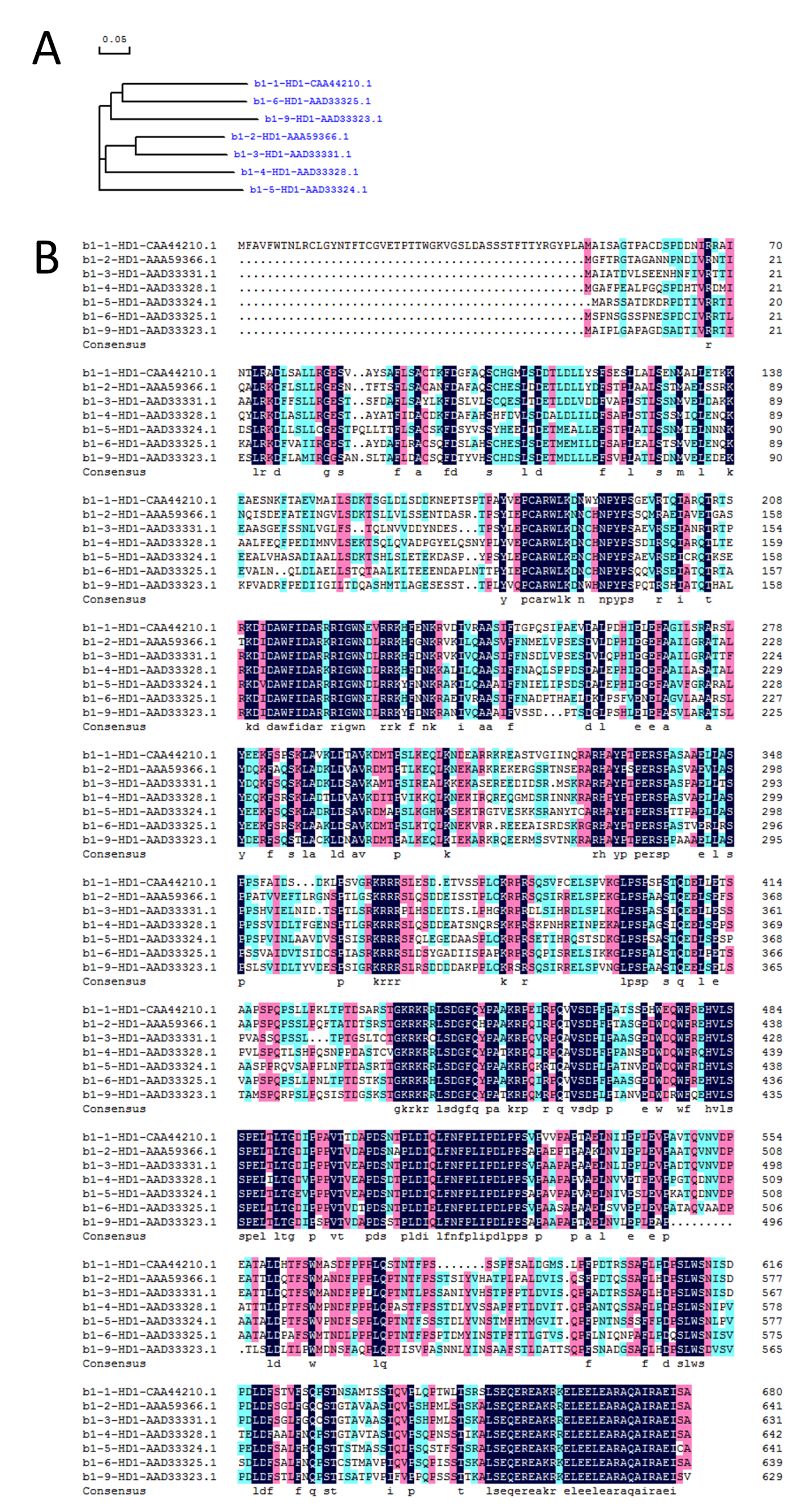

Supplement: Supplemental Material [file supp_g3.116.034637_FigureS15.tif]

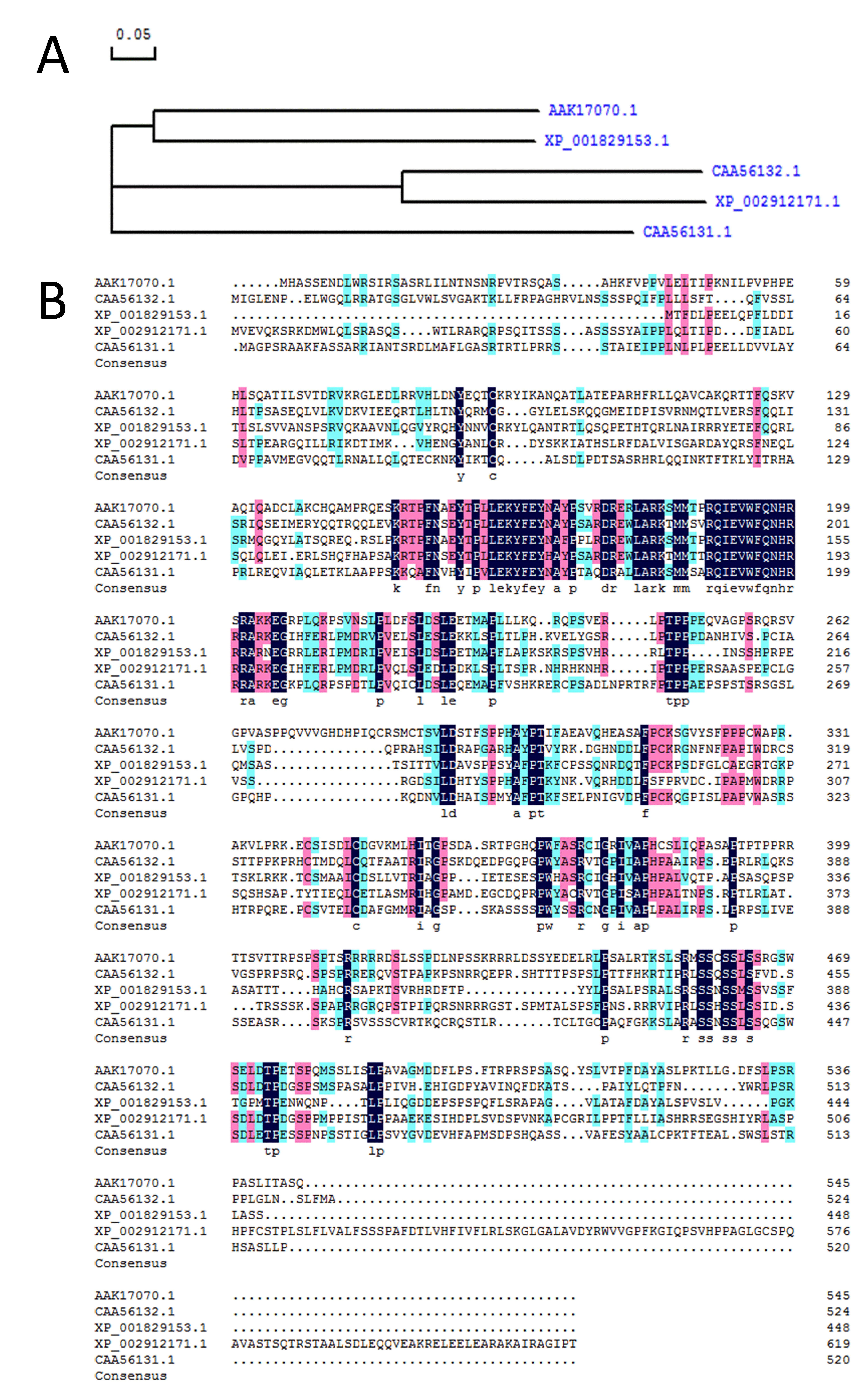

Supplement: Supplemental Material [file supp_g3.116.034637_FigureS16.jpg]

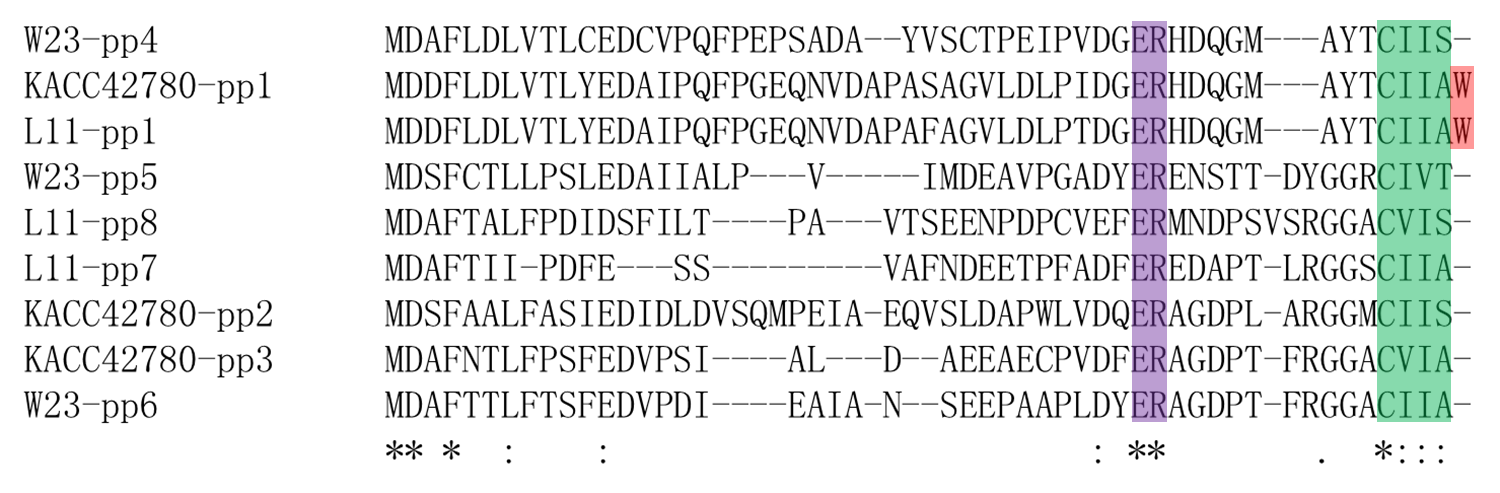

Supplement: Supplemental Material [file supp_g3.116.034637_FigureS17.tif]

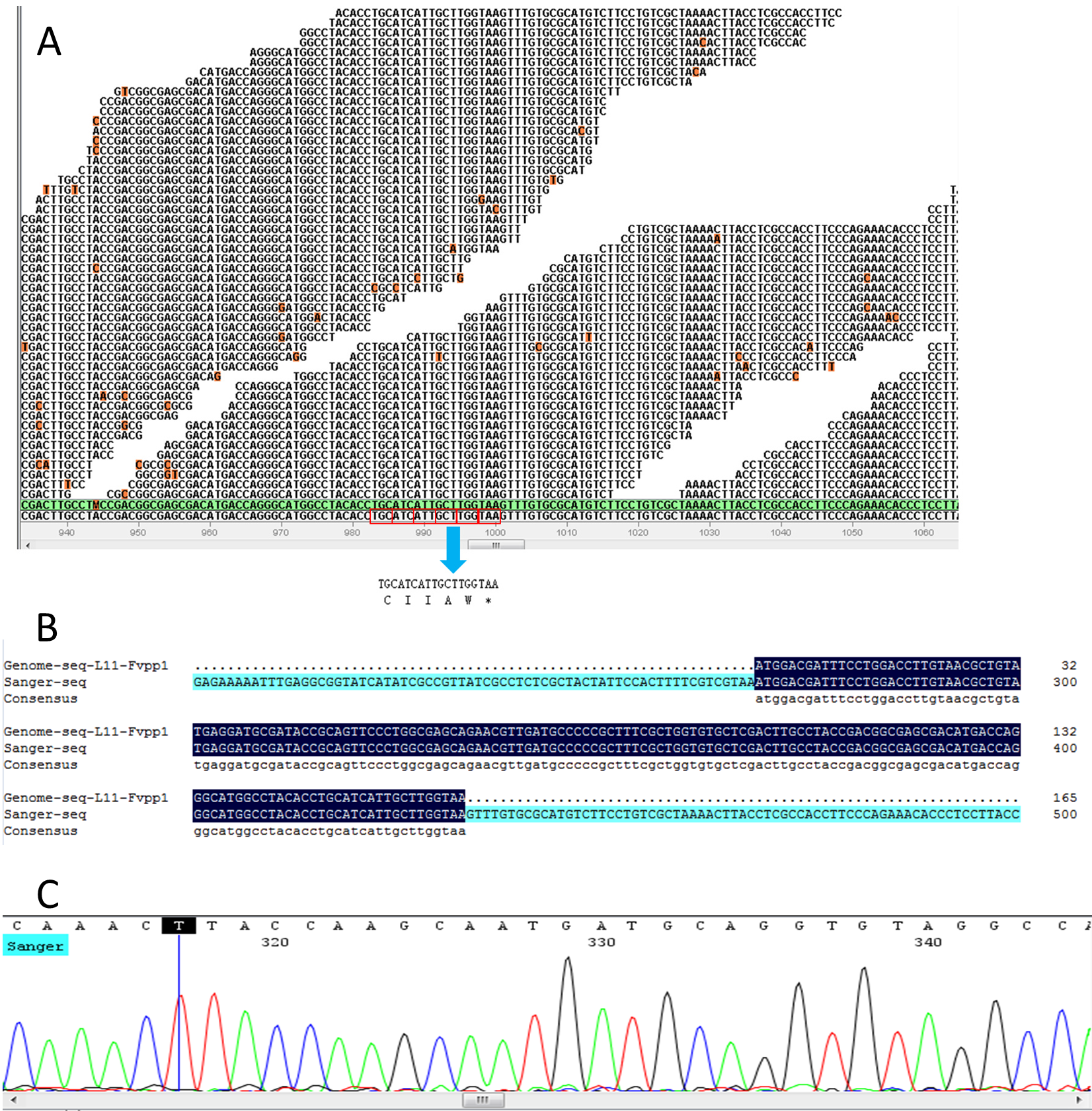

Supplement: Supplemental Material [file supp_g3.116.034637_FigureS18.tif]
